# Continuous vs Routine Electroencephalogram in Critically Ill Adults With Altered Consciousness and No Recent Seizure: A Multicenter Randomized Clinical Trial

**DOI:** 10.1001/jamaneurol.2020.2264

**Published:** 2020-07-27

**Authors:** Andrea O. Rossetti, Kaspar Schindler, Raoul Sutter, Stephan Rüegg, Frédéric Zubler, Jan Novy, Mauro Oddo, Loane Warpelin-Decrausaz, Vincent Alvarez

**Affiliations:** 1 Department of Neurology, Lausanne University Hospital, University of Lausanne, Lausanne, Switzerland; 2 Sleep-Wake-Epilepsy-Center, Department of Neurology, Inselspital, Bern University Hospital and University of Bern, Bern, Switzerland; 3 Clinic for Intensive Care Medicine, University Hospital Basel and University of Basel, Basel, Switzerland; 4 Department of Neurology, University Hospital Basel and University of Basel, Basel, Switzerland; 5 Department of Intensive Care Medicine, Lausanne University Hospital and University of Lausanne, Lausanne, Switzerland; 6 Clinical Trial Unit, Lausanne University Hospital and University of Lausanne, Lausanne, Switzerland; 7 Department of Neurology, Hôpital du Valais, Sion, Switzerland

## Abstract

**Importance:**

In critically ill patients with altered consciousness, continuous electroencephalogram (cEEG) improves seizure detection, but is resource-consuming compared with routine EEG (rEEG). It is also uncertain whether cEEG has an effect on outcome.

**Objective:**

To assess whether cEEG is associated with reduced mortality compared with rEEG.

**Design, Setting, and Participants:**

The pragmatic multicenter Continuous EEG Randomized Trial in Adults (CERTA) was conducted between 2017 and 2018, with follow-up of 6 months. Outcomes were assessed by interviewers blinded to interventions.The study took place at 4 tertiary hospitals in Switzerland (intensive and intermediate care units). Depending on investigators’ availability, we pragmatically recruited critically ill adults having Glasgow Coma Scale scores of 11 or less or Full Outline of Responsiveness score of 12 or less, without recent seizures or status epilepticus. They had cerebral (eg, brain trauma, cardiac arrest, hemorrhage, or stroke) or noncerebral conditions (eg, toxic-metabolic or unknown etiology), and EEG was requested as part of standard care. An independent physician provided emergency informed consent.

**Interventions:**

Participants were randomized 1:1 to cEEG for 30 to 48 hours vs 2 rEEGs (20 minutes each), interpreted according to standardized American Clinical Neurophysiology Society guidelines.

**Main Outcomes and Measures:**

Mortality at 6 months represented the primary outcome. Secondary outcomes included interictal and ictal features detection and change in therapy.

**Results:**

We analyzed 364 patients (33% women; mean [SD] age, 63 [15] years). At 6 months, mortality was 89 of 182 in those with cEEG and 88 of 182 in those with rEEG (adjusted relative risk [RR], 1.02; 95% CI, 0.83-1.26; *P* = .85). Exploratory comparisons within subgroups stratifying patients according to age, premorbid disability, comorbidities on admission, deeper consciousness reduction, and underlying diagnoses revealed no significant effect modification. Continuous EEG was associated with increased detection of interictal features and seizures (adjusted RR, 1.26; 95% CI, 1.08-1.15; *P* = .004 and 3.37; 95% CI, 1.63-7.00; *P* = .001, respectively) and more frequent adaptations in antiseizure therapy (RR, 1.84; 95% CI, 1.12-3.00; *P* = .01).

**Conclusions and Relevance:**

This pragmatic trial shows that in critically ill adults with impaired consciousness and no recent seizure, cEEG leads to increased seizure detection and modification of antiseizure treatment but is not related to improved outcome compared with repeated rEEG. Pending larger studies, rEEG may represent a valid alternative to cEEG in centers with limited resources.

**Trial Registration:**

ClinicalTrials.gov Identifier: NCT03129438

## Key Points

**Question:**

In patients with acute consciousness impairment and no recent seizures, does continuous electroencephalogram (cEEG) correlate with reduced mortality compared with repeated routine EEG (rEEG)?

**Findings:**

In this pragmatic, multicenter randomized clinical trial analyzing 364 adults, cEEG translated into a higher rate of seizures/status epilepticus detection and antiseizure treatment modifications but did not improve mortality compared with rEEG.

**Meaning:**

Pending larger studies, rEEG may represent a valid alternative to cEEG in centers with limited resources.

## Teaser

This randomized clinical trial investigates whether continuous electroencephalogram is associated with reduced mortality compared with routine electroencephalogram.

## Introduction

Electroencephalography (EEG) allows identification of subclinical seizures and status epilepticus (SE) in intensive care unit (ICU) patients,^1,2,3,4^ treatment adjustment under general anesthesia,^5^ is part of prognostication after cardiac arrest,^6,7^ and identifies cerebral ischemia following subarachnoid hemorrhage.^8^ Continuous EEG (cEEG) detects seizure activity^4^ and nonconvulsive SE^9^ more efficiently than routine EEG (rEEG; 20 minutes) and is gaining increasing popularity.^10,11,12,13^ Both European Society of Intensive Care Medicine^1^ and American Clinical Neurophysiology Society^2,3^ suggest cEEG for critically ill patients with altered consciousness.^3^ However, only low-quality evidence supports these recommendations,^1,3^ which may also be difficult to apply in centers lacking human and technical resources.^1,3,12^

There are significant associations between time spent with seizures or SE and worse clinical prognosis in critically ill children^14^ and adults.^15^ Two adult observational studies suggest that cEEG may be associated with better outcome: among 40 000 patients, lower mortality was found in those undergoing cEEG (25%) vs rEEG (39%; adjusted odds ratio [OR], 0.63; 95% CI, 0.52-0.76).^10^ Another observation on 7 million patients showed lower mortality among the 22 000 with cEEG (23%) vs no EEG or rEEG (28%; adjusted OR, 0.83; 95% CI, 0.75-0.92) at the expense of higher costs and hospital length.^12^ Both cross-sectional analyses were retrospective and relied on sampling from discharge diagnoses, implying potential risks of inclusion and information biases, therefore limiting conclusions on causality of associations. Indeed, other studies did not confirm these findings: 234 patients undergoing cEEG had longer hospitalizations and more frequent anticonvulsant prescription modifications but no mortality difference compared with controls without EEG.^16^ Prolonged EEG did not correlate with better outcome in 29 elderly patients with nonconvulsive SE compared with 58 control patients undergoing repeated rEEG.^17^ After cardiac arrest, cEEG prognostic yield seems similar to repeated rEEG,^18^ with no trend toward a different outcome.^19^

Thus, the issue of whether cEEG vs rEEG improves patients’ outcome remains controversial.^20^ This trial’s aim was to evaluate whether cEEG is associated with reduced mortality.

## Methods

### Study Design

This was a Swiss multicenter (Centre Hospitalier Universitaire Vaudois in Lausanne, Hôpital de Sion, Inselspital Bern, and Universitätsspital Basel), pragmatic, randomized clinical trial to evaluate the prognostic yield of cEEG, with nationally coordinated approval by local ethic commissions (project-ID 2017-00268). Inpatients older than 18 years in intensive or intermediate care units having impaired consciousness of any etiology, defined as a Glasgow Coma Scale (GCS) score of 11 or less or a Full Outline of Responsiveness (FOUR) score of 12 or less^21,22^ verified immediately before randomization, referred from the treating team for EEG, were recruited during local investigators’ availability (working hours, not on weekends). Electroencephalogram requests reflected standard clinical practice in the participating hospitals. We excluded patients in palliative care, those risking invasive procedures within 48 hours, and those with recent seizures (36 hours) or SE (96 hours before randomization): it was determined unethical to prevent patients from cEEG to monitor refractory SE treatment. Interventions were started after written approval by a physician unrelated to patient care or the study, then written proxy consent was obtained at 7 ±3 days; written patient’s consent was sought in survivors regaining intellectual capability. Further methodologic details were previously published.^23^ The formal trial protocols can be found in Supplement 1.

### Intervention

Patients were randomized 1:1 through an online program accessible constantly to 1 cEEG or 2 rEEG, stratified by site (eFigure 1 in Supplement 2). Masking to the caring team was impossible owing to the intervention type; however, patients were not aware of the EEG length. All were recorded with video EEG (NicOne; Viasys Neurocare) started within 4 hours after randomization (which occurred immediately after request), using 21 to 23 electrodes following the international 10 to 20 system; reduced montages with at least 11 electrodes were possible in neurosurgical patients,^24,25^ following technical requirements for EEG recordings in this setting.^26^ Continuous EEG lasted 30 to 48 hours; cEEG interruptions less than 2 hours were allowed for diagnostic purposes (eg, neuroimaging). Patients randomized to rEEG had two 20- to 30-minute recordings over 48 hours (no repetition within the same day). Standardized reactivity testing with loud sounds and axial nociceptive stimulations was performed at least twice daily.^27^

All EEG interpreters were certified for the American Clinical Neurophysiology Society Standardized Critical Care EEG Terminology.^28,29,30^ Results were communicated to treating teams within 2 hours of EEG start, at least 3 (working days) or 2 times per day (weekends and bank holidays). A uniform operational definition of electrographic seizures (≥10 seconds) and SE (≥5 minutes) was used: repetitive, rhythmic, or periodic discharges or spike-waves at greater than 3 Hz or at less than 3 Hz with evolution in amplitude, frequency, location, or with electroclinical response to antiseizure drugs (ASD).^4,28,31,32^ The protocolled EEG intervention was stopped in patients diagnosed as having seizures or SE during the intervention period (up to 48 hours); they were subsequently treated according to best practice, allowing conversion to cEEG if needed.

### Variables and Outcomes

We prospectively recorded demographics, estimated modified Rankin Scale (mRS) score before admission, admission reason, comorbidities (Charlson Comorbidity Index [CCI]),^33^ previous epileptic seizures, GCS, or FOUR immediately before EEG intervention, medication during intervention, and adverse events possibly related to intervention. Results are presented according to the Strengthening the Reporting of Observational Studies in Epidemiology (STROBE) reporting guideline.

Mortality at 6 months represented the primary outcome. We assessed secondary outcomes during hospital stay and through semistructured telephone interviews with patients, relatives, or treating physicians^34^ at 4 weeks and 6 months, blinded to intervention. They focused on:

- Midazolam/propofol and ASD prescription at EEG start
- Seizure/SE detection
- Detection of interictal, potentially epileptiform features, including periodic or rhythmic patterns^35^ (excluding generalized anterior rhythmic delta^36^)
- Modification of ASD or sedation (either started, stopped, increased, or decreased), triggered by the EEG results according to treating physicians, occurring over 60 hours following the beginning of the recording; not standardized (pragmatic study)
- Need of additional EEG after the intervention
- Rate of in-hospital infections requiring antibiotics
- Mechanical ventilation duration
- Time to death from randomization
- Hospitalization length in survivors
- mRS; Cerebral Performance Category (CPC)^37^ at 6 months.

We also assessed destination after discharge, ability to return to work, and hospitalization costs; these will be the subject of subsequent studies. This trial was not designed to investigate EEG for delayed ischemia.

### Statistical Analysis

We used Stata, version 14 (StataCorp). The sample size for the primary outcome was calculated^23^ using data available during the study conception: mortality in patients undergoing cEEG patients 14% lower than in those with no cEEG (25% vs 39%)^10^; 2 × 174 patients were needed to detect this difference (2-sided test with 0.8 power; .05 α error; χ^2^ for independent samples). A safety interim analysis of the primary outcome was planned after 100 patients, foreseeing a study interruption if the target difference in primary outcome would have been met: in June 2018, recruitment was continued (cEEG n = 28 of 55; rEEG, n = 27 of 55; *P* = .85, χ^2^).

Analysis of secondary end points^23^ was performed using χ^2^, 2-sided Fisher, *t*, or Mann-Whitney *U* tests as needed. The primary outcome, assessed for patients with available data at 6 months and most relevant secondary outcomes, calculated for all patients with available data, were also assessed with risk ratios (dichotomous variables) and linear regressions (continuous variables). The relative risk (RR) for death at 6 months associated with cEEG was estimated by Poisson regression with robust error variance. For continuous variables, linear regression was performed to calculate coefficients. For exploratory purposes, regressions were adjusted for potential confounders (variables with marked asymmetry across intervention groups despite randomization). We also explored the primary outcome in subgroups of patients with the most prevalent neurologic diagnoses, and patients with deeper consciousness impairment (FOUR ≤10 or GCS ≤8). Therefore, interaction terms were fit to the regression models evaluating relative death risks at 6 months and use of cEEG, to assess effect modification by age, mRS, CCI, hypoxic-ischemic encephalopathy, brain trauma, and intracranial hemorrhage. Significance was set at *P* = .05, with 2-sided approaches.

## Results

**Figure 1.**
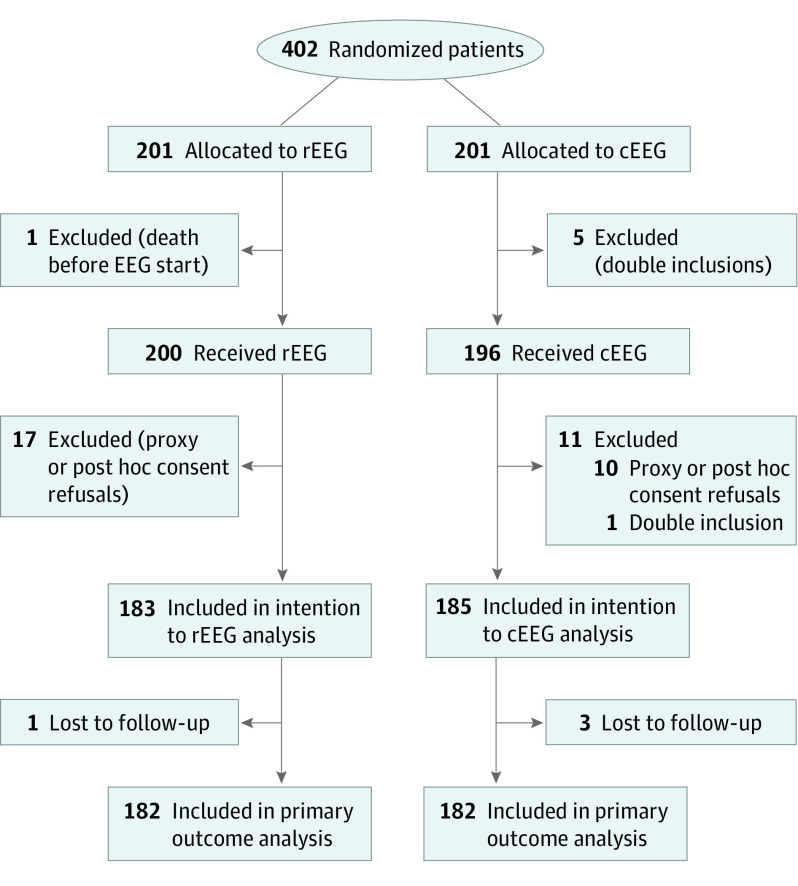
Study Flowchart The number of screened patients was not recorded. cEEG indicates continuous electroencephalogram; rEEG, routine EEG.

Between April 2017 and November 2018, we recruited 402 patients; 6 were excluded before intervention (5 double inclusions and 1 death) and 28 during or shortly thereafter (27 proxy or post hoc consent refusals and 1 double inclusion); 183 participants in the rEEG and 185 in the cEEG were available for outcome assessments. Four patients were lost to follow-up, resulting in 182 participants in each arm available for the primary outcome at 6 months (Figure 1). All patients received the EEG intervention to which they were randomized. No adverse event related to EEG procedures was observed.

Table 1 summarizes baseline characteristics; intervention groups appeared globally balanced, but patients receiving rEEG tended to have a lower burden of comorbidities, less prevalent hypoxic-ischemic encephalopathy and brain trauma, and more prevalent ischemic stroke, toxic metabolic disorders, and other conditions (infections, inflammations, oncological, and/or degenerative). Median EEG duration was in line with the protocol (40 minutes for rEEG [2 × 20 minutes]; 32 hours for cEEG). No relevant difference was observed across centers for demographics, anoxic-ischemic encephalopathy, and time to EEG (data not shown).

**Table 1.** Baseline Patient Characteristics

Characteristic	EEG, No. (%)	
	Routine (n = 183)	Continuous (n = 185)
Female	61 (33.3)	62 (33.5)
Age, mean (SD), y	63.7 (15.3)	63.8 (14.6)
Patient location before hospitalization		
Home	139 (76.0)	147 (79.5)
Other acute hospital	35 (19.1)	33 (17.8)
Rehabilitation clinic or nursing home	9 (4.9)	5 (2.7)
mRS before admission, median (range)	1 (1-5)	1 (1-4)
Reason of admission		
Brain injury (including CA)	102 (55.7)	116 (61.6)
Medical	60 (32.8)	44 (23.8)
Surgical	16 (8.7)	24 (12.4)
Other	5 (2.7)	4 (2.2)
Previous seizures/SE (excluding seizures ≤36 h or SE ≤96 h before randomization)	19 (10.4)	15 (8.1)
SAPS II before EEG intervention, median (range)	50 (8-94)	52 (6-89)
FOUR before EEG intervention, median (range)	4 (0-15)	5 (0-15)
GCS before EEG, median (range)	3 (3-11)	3 (3-11)
CCI before EEG, median (range)	1 (0-10)	1 (0-12)
Patient location during EEG intervention		
Intensive care unit	169 (92.4)	177 (95.7)
Intermediate care unit	11 (6.0)	6 (3.2)
General ward	3 (1.6)	2 (1.1)
Final neurologic diagnosis		
Hypoxic-ischemic encephalopathy	53 (28.9)	60 (32.4)
Brain trauma	17 (9.3)	32 (17.3)
Intracranial hemorrhage	40 (21.9)	47 (25.4)
Ischemic stroke	18 (9.8)	10 (5.4)
Toxic-metabolic, not primarily involving brain	14 (7.7)	9 (4.9)
Other	41 (22.4)	27 (14.6)
Time of EEG after admission, median (range), h	60.3 (1.0-890.0)	57.5 (0.7-2116.7)
EEG duration during intervention, mean (SD), min	40 (9.2)	1925 (792)
ASD administration at first EEG start^a^	56 (30.6)	67 (36.2)
LEV	35 (19.1)	51 (27.6)
VPA	11 (6.0)	13 (7.0)
LCM	5 (2.7)	12 (6.5)
BRV	1 (0.5)	2 (1.0)
PHT	0	2 (1.0)
Propofol administration at first EEG start	95 (51.9)	102 (55.1)
Dose in patients under propofol, median (range), mg/kg/h	1.00 (0.01-4.02)	0.70 (0.01-3.93)
Midazolam administration at 1st EEG start	78 (42.6)	70 (37.8)
Dose in patients under midazolam, median (range), mg/kg/h	0.086 (0.001-2.64)	0.075 (0.001-0.963)

Abbreviations: ASD, antiseizure drug; BRV, brivaracetam; CA, cardiac arrest; CCI, Charlson Comorbidity Index; EEG, electroencephalogram; FOUR, Full Outline of Responsiveness score; GCS, Glasgow Coma Scale score; mRS, modified Rankin Scale; LEV, levetiracetam; LCM, lacosamide; PHT, phenytoin; SAPS II, Simplified Acute Physiology Score; SE, status epilepticus; VPA, valproate.

^a^ Combinations are possible; other ASD included clonazepam, diazepam, gabapentin, ketamine, lamotrigine, lorazepam, oxazepam, perampanel, pregabalin, rufinamide, and topiramate.

**Figure 2.**
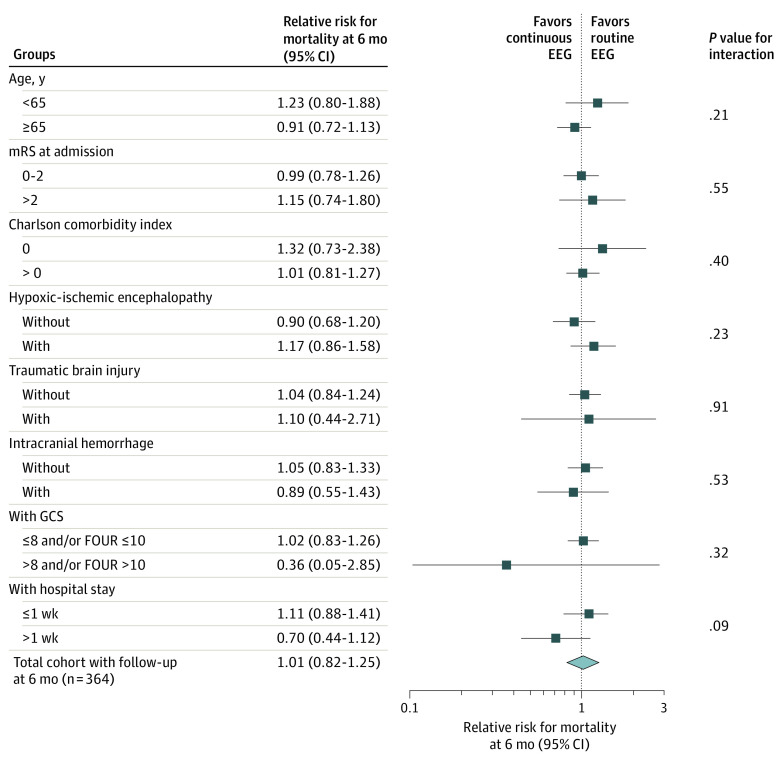
Effect Modification Regarding the Relative Risk of Mortality at 6 Months for Continuous Electroencephalogram (EEG) FOUR indicates Full Outline of Responsiveness score; GCS, Glasgow Coma Scale score; mRS, modified Rankin Scale.

Table 2 illustrates outcomes at 6 months. Mortality did not differ across intervention groups and centers (Le Centre Hospitalier Universitaire Vaudois: n = 147 of 287; other hospitals, n = 40 of 97; *P* = .91; χ^2^). This did not change after adjusting for CCI and hypoxic-ischemic encephalopathy nor after exploratory stratification for hypoxic-ischemic encephalopathy (eTable in Supplement 2), or age, baseline mRS, CCI, traumatic brain injury, intracranial hemorrhage, severity of consciousness impairment, or time to EEG (Figure 2). Limiting analysis to survivors, CPC did not change across groups, while mRS evolution at 6 months was better in the rEEG group, especially in patients without hypoxic-ischemic encephalopathy; eFigure 2 in Supplement 2 shows the distribution of functional outcomes.

**Table 2.** Primary Outcome (Mortality at 6 Months) and Functional Outcomes Across cEEG vs rEEG (Poisson Regression Models for Categorical Variables [Mortality] and Linear Regression Models for Continuous Variables [Δ mRS and CPC])^a^

Outcome	rEEG (n = 182), No. (%)	cEEG (n = 182), No. (%)	Crude		Adjusted for CCI, cardiac arrest	
			Relative risk (95% CI)	*P* value	Relative risk (95% CI)	*P* value
Mortality at 6 mo, No. (%)	88 (48.4)	89 (48.9)	1.01 (0.82 to 1.25)	.92	1.02 (0.83 to 1.26)	.85
	**Median (range)**	**Median (range)**	**Regression coefficient**	***P* value**	**Regression coefficient**	***P* value**
Δ mRS at 6 mo, survivors	1 (−5 to 4)	1 (−3 to 5)	0.65 (0.13 to 1.16)	.01	0.63 (0.13 to 1.14)	.01
CPC at 6 mo, survivors	2 (1 to 4)	2 (1 to 4)	0.08 (−0.17 to 0.34)	.52	0.08 (−0.18 to 0.33)	.55

Abbreviations: CCI, Charlson Comorbidity Index; cEEG, continuous electroencephalography; CPC, cerebral performance category; Δ mRS, evolution of modified Rankin Scale between prehospitalization and at 6 months; rEEG, routine electroencephalography.

^a^ Results given as crude, and adjusted for hypoxic-ischemic encephalopathy and CCI. *P *values less than .05 are significant.

Table 3 outlines exploratory secondary outcomes according to intention-to-monitor during the EEG intervention (if an rEEG was converted to cEEG and the patient quit intervention; results of the latter and subsequent treatment were not counted for this analysis). Detection of features of ictal-interictal continuum, seizures, and/or SE were more frequent in the cEEG group, as was the modification rate of ASD prescription; conversely, sedatives, need for EEG after intervention, infections, mechanical ventilation duration, time to death since randomization, and survivors’ length of stay did not differ. To explore whether cEEG facilitated decisions of life-sustaining treatment withdrawal, we further analyzed death latency, which was comparable in the subgroup with hypoxic-ischemic encephalopathy (rEEG: median, 8.5 days; range, 0-156; cEEG: median, 6 days; range, 0-157; *P* = .07) and without (rEEG: median, 11 days; range, 1-130; cEEG: median, 8 days; range 1-176; *P* = .40; *U* tests).

**Table 3.** Exploratory Analyses: Associations of Secondary Outcome Measures With EEG Type^a^

Outcome	No. (%)		Relative risk (95% CI)	*P* value
	rEEG (n = 183)	cEEG (n = 185)		
Features of ictal-interictal continuum detected, without seizures/SE	102 (55.7)	128 (69.2)	1.24 (1.06-1.46)	.009
Seizures/SE detected	8 (4.4)	29 (15.7)	3.59 (1.68-7.64)	.001
Changes in antiseizure drug prescription within 60 h following start of EEG intervention^b^	21 (11.5)	39 (21.1)	1.84 (1.12-3.00)	.01
Changes in sedation prescription within 60 h following start of EEG intervention^b^	8 (4.4)	13 (7.0)	1.61 (0.683-3.79)	.27
Need of additional EEG after intervention	41 (22.8)^c^	56 (31.1)	1.37 (0.97-1.93)	.08
In-hospital infection requiring antibiotics	56 (30.8)	47 (25.7)	0.82 (0.61-1.11)	.20
Length of ventilation need, median (range), h	123 (0-837)	138 (0-1214)	NA	.47
Length of hospital stay in survivors, median (range), d	25.3 (2.6-393.3)	24.5 (1.4-161.1)	NA	.84
Time to death since randomization, median (range), d	8.5 (0-157)	6 (0-176)	NA	.07

Abbreviations: cEEG, continuous electroencephalography; NA, not applicable; rEEG, routine electroencephalography; SE, status epilepticus.

^a^ Relative risk for categorical variables (ictalinterictal continuum, seizures/se, changes in antiseizure drug or sedation treatment, need of additional EEG, or in-hospital infection) and *U* test for continuous variables (duration of ventilation support, length of stay in survivors, or time to death). Values less than .05 are significant.

^b^ Motivated by EEG results according to treating physicians.

^c^ Including 5 (2.7%) converted to cEEG.

## Discussion

To our knowledge, this represents the first randomized clinical trial in this clinical setting. It reveals similar long-term mortality in critically ill adults with altered consciousness without recent seizures randomized to repeated rEEG or cEEG, despite higher detection of ictal and interictal EEG features and ASD modifications in those receiving cEEG.

Patients were recruited following a quantitative definition of consciousness impairment, unlike previous observations.^10,12,16^ Additionally, they were assessed for comorbidities and estimated disability before intervention, allowing evolution characterization over 6 months. This represents a reasonable estimation of long-term outcome, unlike mortality at discharge reported previously.^10,12,16^ In our view, the principal finding is corroborated by adjustment for imbalances in baseline characteristics despite randomization, exploration of potential effects of demographics and etiologies, and comparable latency to death in both interventions. Median cEEG recording (32 hours) may appear shorter than the usual duration in some centers, but is longer than a retrospective evaluation from 3 large US hospitals^38^ and in line with a 2018 Dutch survey^13^; as opposed to that assessment, all our recordings had concomitant video. Notably, the 2 discharge-based studies did not report on cEEG duration^10,12^ and 93% of seizures seem to be detectable within 24 to 48 hours.^4^

Overall mortality (48.6%) was higher than in the 2 retrospective observations (22% and 39%^10,12^), possibly reflecting enrollment of 30.7% hypoxic-ischemic patients, a condition related to 50% mortality. Also, exclusion from analysis (per Swiss law) of survivors subsequently withdrawing patient consent inflated our mortality rate (mortality would have been 45.4% considering these 26 patients). Because previous studies do not detail on diagnoses, direct comparisons are impossible. Additionally, those studies assessed mortality at discharge, potentially underestimating it at 6 months. Finally, a randomized study appears different from retrospective assessments of discharge diagnoses.

Mortality in patients without cardiac arrest showed a nonsignificant trend favoring cEEG: the absolute difference of 4.5% lies at less than the targeted 14% but is similar to the 5% reported in the 2019 retrospective assessment.^12^ This might orient on the sample size needed for a future trial, where a difference of 4% (for example, a decrease from 48% to 44%) would imply enrollment of 4872 analyzable patients without cardiac arrest (2-sided proportion test; *P* = .05; power = 0.8). The fact that disability (mRS) evolved more favorably in survivors without cardiac arrest undergoing rEEG seems to counterbalance the mortality tendency.

This trial confirms that cEEG leads to increased detection of ictal and interictal EEG features than rEEG, even excluding patients with recent seizures or SE. The rEEG detection rate (4.4% over 40 minutes) represents roughly one-quarter of cEEG (15.7% over 32 hours in median); the latter is comparable with literature data from the past few years (2017-2019),^38,39^ also including patients after cardiac arrest, and suggests reasonable generalizability of our findings. The observational cEEG study identified ictal changes in 12.3% to 13.6%,^38^ the meta-analysis in 6.3% of rEEG and 15.6% of cEEG.^39^ The pooled prevalence of repetitive or rhythmic features of the ictal-interictal continuum, excluding generalized rhythmic delta, was between 28% to 29% in 3 US centers^38^ and 4% to 9% for rEEG and 7% to 15% for cEEG in the meta-analysis.^39^ These broad proportions appear lower than ours (55.7% for rEEG and 69.2% for cEEG), possibly following inclusion of sporadic epileptiform features in our labeling, and maybe higher assessment accuracy; all our interpreters were American Clinical Neurophysiology Society certified, strengthening external validity.

One-third of patients received ASD at EEG start, reflecting prophylaxis following brain trauma and the fact that about 10% of participants had previous seizures (before the timeframe defined as exclusion criterium). Antiseizure drug prescription was modified more frequently in the cEEG arm, likely following the refined diagnosis of interictal and ictal changes compared with rEEG. The modification rate (21%; 28% if including changes in sedation) appears lower than reported in previous observational studies.^16,40^ However, those modifications occurred during the entire hospitalization, as opposed to our time restriction over the first 60 hours after EEG start and the definition of association with EEG results, which should better (although more conservatively) reflect the effect of the EEG intervention.

Improved diagnostics and increased modification in ASD do not seem to translate into better clinical outcome (not only mortality but also functional) nor different hospitalization length in survivors. One possible explanation is that EEG may trigger decisions to life-sustaining treatment withdrawal. This would rather involve both arms (background is the most informative feature in this context^25^). Mortality and mRS evolution in survivors were actually not different between intervention groups considering patients with cardiac arrest (where EEG is an integral part of these decisions^41^); furthermore, previous studies did not show any mortality difference in this particular diagnosis across EEG durations.^19,42^ Also, although unfortunately we do not have information on death causes, death latency was relatively similar across EEG types in the whole cohort (median: 1 week after intervention); a massive effect of active withdrawal seems unlikely. Another potential explanation may involve underlying cerebral structural damage independently from the additional role played by epileptic phenomena. As illustrated for patients with SE, successful treatment of electrical dysfunction may be futile if the effects of initial structural injury are predominant.^43,44^ This might suggest that underlying variables not related to epileptic aspects may represent additional important determinants of prognosis in this setting.

Need of subsequent EEG after the intervention was not significantly higher in cEEG, possibly reflecting a higher seizure and SE detection rate. Sedation at baseline was comparable across groups and globally given at relatively low dosage, and EEG-triggered changes were minor; duration of mechanical ventilation did not differ.

### Limitations

This study has limitations. Our sample size is based on the only available comparison at the time of design (2015-2016), and analysis of several secondary outcomes may have been underpowered. We enrolled patients with hypoxic-ischemic encephalopathy having a high mortality risk.^45^ However, they represent one of the most frequent ICU neurologic diagnoses,^46^ and some previous observational cEEG studies also included them.^10,39^ As mentioned, a considerably larger patient sample without this condition may allow detecting small outcome differences. We did not record the start of altered consciousness, but EEG was performed within 4 hours after request (see Methods). The time of EEG intervention since hospital admission may seem relatively long, but restricting analysis to patients admitted less than a week ago does not change the results. We pragmatically studied a referral cohort. There was unfortunately no screening of all potential candidates (recruitment occurred only during working hours); we recognize that this may represent a selection bias. In addition, many patients received sedation at baseline, which may reduce seizure detection, but doses were globally low and comparable across groups. Although suboptimal, this reflects clinical routine; the similarities of our cEEG seizure detection rates to previous studies seem to corroborate our findings’ generalizability. The cohort is heterogeneous in terms of etiologies, but inclusion criteria closely fit current recommendations. We assessed the relation of mortality with a diagnostic test, not a treatment. However, EEG results were provided regularly and timely, translating into changes in clinical management. We excluded patients having seizures or SE immediately before enrollment, potentially lowering the EEG yield, but it seems that cEEGs ordered to monitor already-diagnosed SE represent a minority of requests in clinical practice.^38^ Our findings are not generalizable to ICU patients with incident seizures or SE, in whom cEEG is commonly used for treatment monitoring. The protocol foreseeing communication to the caregivers several times per day, and recording durations of at least 30 hours, corresponds to clinical practice and actually lies beyond current recommendations.^2^

## Conclusions

Considering these limitations, despite increased detection rates of interictal and ictal features and of EEG-driven modification of antiseizure therapy, cEEG does not seem to correlate with improved patient outcome compared with repeated rEEG. Pending larger studies in a more homogeneous patient population, repeated rEEG may represent a reasonable alternative to cEEG, at least in centers with limited resources.

## Notes

Supplement 1. Trial Protocol

Supplement 2. **eTable 1.** Exploratory analysis of primary outcome (mortality at 6 months) and functional outcomes **eFigure 1.** Study design **eFigure 2.** Cerebral performance categories (CPC) and modified Rankin Scales (mRS) at 6 months in the two intervention groups

Supplement 3. Data Sharing Statement
