# Supplementary material for: Continuous vs Routine Electroencephalogram in Critically Ill Adults With Altered Consciousness and No Recent Seizure: A Multicenter Randomized Clinical Trial
Source: JAMA Neurol. 2020 Jul 27;77(10):1–8. doi: 10.1001/jamaneurol.2020.2264 (PMC7385681; doi:10.1001/jamaneurol.2020.2264)
Supplement: Supplement 1. — Trial Protocol [file jamaneurol-e202264-s001.pdf]

Département neurosciences cliniques
Service de neurologie
BH-07
Rue du Bugnon 46
CH-1011 Lausanne

Clinical Study Protocol

Impact on clinical outcome of continuous video-electroencephalography (cEEG) monitoring in patients with disorders of consciousness: A randomized controlled trial

__C__ontinuous __E__EG __R__andomized __T__rial in __A__dults
"CERTA"

Study Type:	"Other Clinical Trial" as per ClinO
Study Categorisation:	Risk category A
Study Registration:	www.clinicaltrial.gov registry and FOPH portal
Study Identifier:	CERTA
Sponsor, Sponsor-Investigator or Principal Investigator:	Centre Hospitalier Universitaire Vaudois (CHUV)<br>Prof Andrea Rossetti<br>Service de neurologie<br>Rue du Bugnon 46, BH07<br>1011 Lausanne<br>Tel : 0041.79.556.84.15<br>andrea.rossetti@chuv.ch
Investigational Product:	None
Protocol Version and Date:	V2.0 - 29.05.2017

CONFIDENTIAL
The information contained in this document is confidential and the property of the sponsor. The information may not - in full or in part - be transmitted, reproduced, published, or disclosed to others than the applicable Competent Ethics Committee(s) and Regulatory Authority(ies) without prior written authorisation from the sponsor, except to the extent necessary to obtain informed consent from those who will participate in the study.

Signature Pages (1/5)

Study number	www.clinicaltrial.gov Nr. NCT03129438 and FOPH portal Nr. *tbd*
Study Title	**Impact on clinical outcome of continuous video-electroencephalography (cEEG) monitoring in patients with disorders of consciousness: a randomized controlled trial**

The sponsor-coordinating investigator and trial statistician have approved the protocol version **V2.0 dated 29.05.2017**, and confirm hereby to conduct the study according to the protocol, current version of the World Medical Association Declaration of Helsinki, ICH-GCP guidelines and the local legally applicable requirements.

Sponsor-coordinating investigator:
Prof Andrea Rossetti

Lausanne, 29/5/2017

Place/Date

Signature

Statistician:
PD Dr. Raoul Sutter

Basel, 15/6/2017

Place/Date

Signature

Signature Pages (2/5)

Study number	www.clinicaltrial.gov Nr. NCT03129438 and FOPH portal Nr. *tbd*
Study Title	**Impact on clinical outcome of continuous video-electroencephalography (cEEG) monitoring in patients with disorders of consciousness: a randomized controlled trial**

Local Principal Investigator at study site:

I have read and understood this trial protocol and agree to conduct the trial as set out in this study protocol, the current version of the World Medical Association Declaration of Helsinki, ICH-GCP guidelines and the local legally applicable requirements.

Site	Hôpital du Valais, Sion, CH
Principal investigator	Dr Vincent Alvarez

Dr Vincent Alvarez
Médecin adjoint
Service de neurologie
Hôpital du Valais
Hôpital de Sion
1950 Sion

Sion, May 29th 2017
Place/Date

Signature

Signature Pages (3/5)

Study number	www.clinicaltrial.gov Nr. NCT03129438 and FOPH portal Nr. *tbd*
Study Title	**Impact on clinical outcome of continuous video-electroencephalography (cEEG) monitoring in patients with disorders of consciousness: a randomized controlled trial**

Local Principal Investigator at study site:

I have read and understood this trial protocol and agree to conduct the trial as set out in this study protocol, the current version of the World Medical Association Declaration of Helsinki, ICH-GCP guidelines and the local legally applicable requirements.

Site	Universitätsspital Basel, Basel, CH
Principal investigator	Pr Stephan Rueegg

Universitätsspital Basel
Abteilung für klinische Neurophysiologie
Neurologische Klinik
Prof. Dr. med. St. Rüegg
Leitender Arzt
CH-4031 Basel

Basel, 29/05/2017

Place/Date

Signature

Signature Pages (4/5)

Study number	www.clinicaltrial.gov Nr. NCT03129438 and FOPH portal Nr. *tbd*
Study Title	**Impact on clinical outcome of continuous video-electroencephalography (cEEG) monitoring in patients with disorders of consciousness: a randomized controlled trial**

Local Principal Investigator at study site:

I have read and understood this trial protocol and agree to conduct the trial as set out in this study protocol, the current version of the World Medical Association Declaration of Helsinki, ICH-GCP guidelines and the local legally applicable requirements.

Site	Inselspital, Bern, CH
Principal investigator	Pr Kaspar Schindler

Bern 29/5/17

Place/Date

Signature

Signature Pages (5/5)

Study number	www.clinicaltrial.gov Nr. NCT03129438 and FOPH portal Nr. *tbd*
Study Title	**Impact on clinical outcome of continuous video-electroencephalography (cEEG) monitoring in patients with disorders of consciousness: a randomized controlled trial**

Local Principal Investigator at study site:

I have read and understood this trial protocol and agree to conduct the trial as set out in this study protocol, the current version of the World Medical Association Declaration of Helsinki, ICH-GCP guidelines and the local legally applicable requirements.

Site	Hôpitaux Universitaires de Genève (HUG), Genève, CH
Principal investigator	Pr Margitta Seeck

Place/Date ______________________ Signature ______________________

Table of Contents

STUDY SYNOPSIS **10**
STUDY SUMMARY IN LOCAL LANGUAGE **13**
ABBREVIATIONS **14**
STUDY SCHEDULE **15**
1. STUDY ADMINISTRATIVE STRUCTURE **16**
1.1 Sponsor, Sponsor-Investigator 16
1.2 Principal Investigator(s) 16
1.3 Statistician ("Biostatistician") 17
1.4 Laboratory 17
1.5 Monitoring institution 17
1.6 Data Safety Monitoring Committee 18
1.7 Any other relevant Committee, Person, Organisation, Institution 18
2. ETHICAL AND REGULATORY ASPECTS **19**
2.1 Study registration 19
2.2 Categorisation of study 19
2.3 Competent Ethics Committee (CEC) 19
2.4 Competent Authorities (CA) 19
2.5 Ethical Conduct of the Study 19
2.6 Declaration of interest 19
2.7 Patient Information and Informed Consent 20
2.8 Participant privacy and confidentiality 21
2.9 Early termination of the study 21
2.10 Protocol amendments 22
3. BACKGROUND AND RATIONALE **23**
3.1 Background and Rationale 23
3.2 Investigational Product (treatment, device) and Indication 24
3.3 Preclinical Evidence 24
3.4 Clinical Evidence to Date 24
3.5 Dose Rationale / Medical Device: Rationale for the intended purpose in study (pre-market MD)25
3.6 Explanation for choice of comparator (or placebo) 25
3.7 Risks / Benefits 25
3.8 Justification of choice of study population 25
4. STUDY OBJECTIVES **26**
4.1 Overall Objective 26
4.2 Primary Objective 26
4.3 Secondary Objectives 26
4.4 Safety Objectives 26
5. STUDY OUTCOMES **27**
5.1 Primary Outcome 27
5.2 Secondary Outcomes 27
5.3 Other Outcomes of Interest 27
5.4 Safety Outcomes 27
6. STUDY DESIGN **28**
6.1 General study design and justification of design 28
6.2 Methods of minimising bias 28
6.2.1. Randomisation 28

6.2.2. Blinding procedures 28
6.2.3. Other methods of minimising bias 28
6.3 Unblinding Procedures (Code break) 28
7. STUDY POPULATION 29
7.1 Eligibility criteria 29
7.2 Recruitment and screening 29
7.3 Assignment to study groups 29
7.4 Criteria for withdrawal / discontinuation of participants 29
8. STUDY INTERVENTION 30
8.1 Identity of Investigational Products (treatment / medical device) 30
Experimental Intervention 30
Control Intervention 30
Packaging, Labelling and Supply (re-supply) 30
Storage Conditions 30
8.2 Administration of experimental and control interventions 30
Experimental Intervention 30
Control Intervention 30
8.3 Dose / Device modifications 30
8.4 Compliance with study intervention 31
8.5 Data Collection and Follow-up for withdrawn participants 31
8.6 Trial specific preventive measures 31
8.7 Concomitant Interventions (treatments) 31
8.8 Study Drug / Medical Device Accountability 31
8.9 Return or Destruction of Study Drug / Medical Device 31
9. STUDY ASSESSMENTS 32
9.1 Study flow charts 32
9.2 Assessments of outcomes 33
9.2.1. Assessment of primary outcome 33
9.2.2. Assessment of secondary outcomes 33
9.2.3. Assessment of other outcomes of interest 33
9.2.4. Assessment of safety outcomes 33
9.2.5. Assessments in participants who prematurely stop the study 33
9.3 Procedures at each visit 33
Screening and enrolment study period – Day 1 (T-3 to T0) 34
Intervention study period – Day 1 to Day2/3 (T0 to T48) 34
Follow up at Day 3/4, T48 to T60 hours 34
Follow up at Week 4 34
Follow up at Month 6 34
10. SAFETY 35
10.1 Interventions studies 35
10.1.1. Definition, assessment and reporting of (serious) adverse events and other safety related events 35
10.1.2. Follow up of (Serious) Adverse Events 35
11. STATISTICAL METHODS 36
11.1 Hypothesis 36
11.2 Determination of Sample Size 36
11.3 Statistical criteria of termination of trial 36

11.4 Planned Analyses 36
11.4.1. Datasets to be analysed, analysis populations 37
11.4.2. Primary Analysis 37
11.4.3. Secondary Analyses 37
11.4.4. Interim analyses 37
11.4.5. Safety analysis 37
11.4.6. Deviation(s) from the original statistical plan 37
11.5 Handling of missing data and drop-outs 37
12. QUALITY ASSURANCE AND CONTROL 38
12.1 Data handling and record keeping / archiving 38
12.1.1 Case Report Forms 38
12.1.2. Specification of source documents 38
12.1.3. Record keeping / archiving 38
12.2 Data management 38
12.2.1. Electronic Data Capture (EDC) system and underlying database 38
12.2.2. Data entry, and validation process 38
12.2.3. Electronic and central data validation 39
12.2.4. Analysis and archiving 39
12.2.5. Data sharing plan 39
12.3 Monitoring 39
12.4 Audits and Inspections 40
12.5 Confidentiality, Data Protection 40
12.6 Storage of biological material and related health data 40
13. PUBLICATION AND DISSEMINATION POLICY 41
14. FUNDING AND SUPPORT 42
14.1 Funding 42
14.2 Other Support 42
15. INSURANCE 43
16. REFERENCES 44
17. APPENDICES 46

STUDY SYNOPSIS

**Sponsor / Sponsor-Investigator:**	Centre Hospitalier Universitaire Vaudois (CHUV) - Prof Andrea Rossetti, MD<br>(*investigator-initiated trial*)
**Study Title:**	Impact on clinical outcome of continuous video-encephalography (cEEG) monitoring in patients with disorders of consciousness: a randomized controlled trial
**Short Title / Study ID:**	CERTA
**Protocol Version and Date:**	V2.0 dated 29.05.2017
**Trial registration:**	www.clinicaltrial.gov Nr. NCT03129438<br>Federal Office of Public Health's (FOPH) portal Nr. *tbd*
**Study category and Rationale**	Risk category A, as:<br>-The health-related intervention investigated entails only minimal risks and burdens<br>-The health-related intervention investigated is recommended by the European Society of Intensive Care Medicine and the American Clinical Neurophysiology Society for most patients with consciousness disorders.
**Clinical Phase:**	NA
**Background and Rationale:**	Continuous video-electroencephalography monitoring (cEEG), a non-invasive tool to monitor electrical brain function, improves seizure detection in comatose patients in intensive care units (ICUs). It is thus recommended for most patients with acute consciousness disorders. cEEG is however resource consuming compared to routine video-EEG (rEEG, lasting 20-30 minutes). While US centers have been using cEEG increasingly, all Swiss hospitals still do not have enough resources to comply with these guidelines. In addition, only one population-based study based on discharge diagnoses suggested that cEEG may improve patients' outcome. Current guidelines are thus based upon weak evidence and expert opinions: whether cEEG leads to improved patients' care remains elusive. Finally, little attention has been drawn towards quantitative EEG information beyond visual analysis, and the impact of such information on diagnosis, treatment, and outcome.
**Objective(s):**	To assess if cEEG in patients with consciousness impairment is related to better functional outcome as compared to rEEG, and to address the prognostic role of quantitative network EEG analyses in this cohort.
**Outcome(s):**	<u>Primary outcome:</u> mortality at 6 months.<br><u>Secondary outcomes:</u> functional outcome at 4 weeks and 6 months, seizure/status epilepticus (SE) detection rate and time to detection, infections rate, duration of intensive care unit stay, change in patient management (antiepileptic drug introduced, increased, or stopped, brain imaging), and costs.
**Study design:**	Multicenter, open label, randomized, active controlled trial.

**Inclusion / Exclusion criteria:**	Inclusion:<br>• In-patients aged ≥ 18 years, treated in an ICU or intermediate care unit.<br>• Alteration of mental state of any etiology (i.e., primarily cerebral or not), with Glasgow-coma scale ≤11 or FOUR score ≤ 12.<br>• Need of an EEG to exclude seizures or SE, or to evaluate prognosis as per the treating physician or the consulting neurologist.<br>• Informed consent obtained for research in emergency situation according to Human Research Act (HRA) art 30-31 at the time of inclusion.<br><br>Exclusion:<br>• Clinical and/or electrographic *status epilepticus* < 96h before randomization<br>• Clinical and/or electrographic seizure < 36h before randomization<br>• Palliative care situation, in which detection of SE or seizures would not have any impact on the patient's care.<br>• High likelihood of needing a surgical intervention or invasive diagnostic procedure within the next 48 hours.
**Measurements and procedures:**	Eligible patients will receive cEEG or rEEG in the first 48 hours following 1:1 randomization.<br><br>Demographics, etiology, Charlson Comorbidity Index, diagnosis leading to EEG, need and length of mechanical ventilation, and subsequent use of rEEG/cEEG will be prospectively collected. Outcomes will be assessed at 4 weeks and 6 months.<br><br>Analyses will compare the two interventional groups (intention to monitor, according to intervention allocation) regarding the outcomes. Additionally, lope cross correlation and horizontal visibility graphs will be applied to compute a weighted adjacency matrix consisting of pairwise interdependences between EEG signals, to characterize the integrative and segregative characteristics of the underlying functional brain networks and compare their relationship with the primary outcome.
**Study Product / Intervention:**	Continuous video-EEG performed once for 30-48 hours.
**Control Intervention (if applicable):**	Routine video-EEG performed 2 times (20-30 min each) within 30-48 hours.
**Number of Participants with Rationale:**	According to a previous estimate, patients with consciousness disorders undergoing cEEG have a 75% survival rate; while patients without cEEG 61%. Using a power of 0.8, an α error of 0.05, and a 2-side approach, 2x174 patients would be needed to detect this significant difference in survival (primary outcome).
**Study Duration:**	30 months
**Study Schedule:**	Planned First Patient First Visit (FPFV): April 2017<br>Planned Last Patient Last Visit (LPLV): September 2019
**Investigator(s):**	Coordinating investigator: Prof Andrea Rossetti, CHUV- Service de neurologie BH07, 1011 Lausanne
**Study Centre(s):**	Multi-centre study in Switzerland:<br>• CHUV, Lausanne (coordinating site)<br>• Hôpital du Valais, Sion<br>• Universitätsspital, Basel<br>• Inselspital, Bern<br>• Hôpitaux Universitaires de Genève (HUG), Genève

**Statistical Considerations:**	Patients with consciousness disorders undergoing cEEG have a 75% survival rate; while patients without cEEG 61%. Using a power of 0.8, an α error of 0.05, and a 2-side approach, 2x174 patients would be needed to detect this significant difference in survival.<br><br>At study completion, the two interventional groups will be compared regarding survival at six months as "intention to monitor" (predefined analysis for the primary endpoint) and "per protocol", adjusted for potential confounders (logistic regressions).<br><br>Analysis of each secondary endpoint will be also conducted using univariable and multivariable approaches.
**GCP Statement:**	This study will be conducted in compliance with the protocol, the current version of the Declaration of Helsinki, the ICH-GCP as well as all national legal and regulatory requirements.

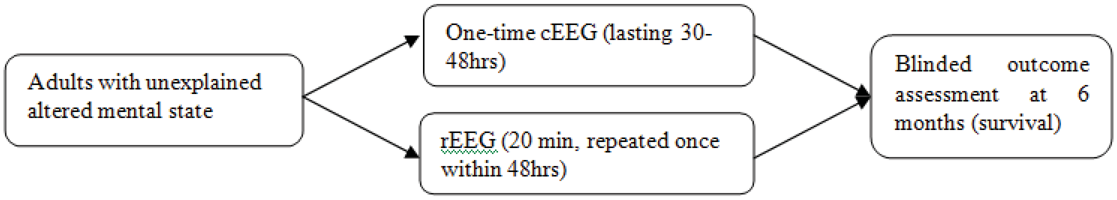

***Synopsis 1**: representative illustration of the flow of the study. cEEG=continuous EEG; rEEG=routine EEG.*

STUDY SUMMARY IN LOCAL LANGUAGE

Résumé en français :

La surveillance par électroencéphalogramme-vidéo continu (cEEG) est un outil non-invasif pour surveiller l'activité électrique cérébrale chez les patients avec atteinte de la conscience. Le cEEG améliore la détection de crises épileptiques. Cependant, l'impact sur le pronostic clinique n'a pas été clairement étudié. Le cEEG implique du temps et des ressources, comparé à l'électroencéphalogramme-vidéo de routine (rEEG, qui dure typiquement 20-30 minutes). Si en Amérique du Nord il est utilisé de manière croissante, la vaste majorité des centres européens n'ont pas les ressources pour une implémentation. De plus, le rôle de l'analyse quantitative du signal EEG, au delà de l'analyse visuelle, concernant le pronostic reste indéterminé.

Le but de cette étude est de déterminer si le cEEG est corrélé à une amélioration du pronostic clinique chez des patients avec une atteinte de la conscience, et d'explorer le rôle pronostique de l'analyse quantitative de l'EEG.

Dans cet essai randomisé contrôlé multicentrique (5 hôpitaux suisses), des patients adultes avec atteinte de la conscience et nécessitant un EEG seront randomisés 1 :1 vers un cEEG durant 30-48 heures ou vers 2 rEEG dans le même lapse de temps. La mortalité à 6 mois représentera l'outcome primaire. Selon une estimation préalable, il faudra 350 patients pour démontrer une différence significative. Les outcomes secondaires seront, entre autres : le devenir fonctionnel, la proportion de crises épileptiques détectées, et les coûts. De même, des analyses quantitatives du signal seront effectuées sur les tracés cEEG et rEEG et corrélées au pronostic.

Cette étude clarifiera si le cEEG a un impact significatif et notable sur le pronostic clinique, définira son efficacité économique, et identifiera les analyses quantitatives du signal EEG corrélées au pronostic. Ses résultats ont le potentiel de générer un impact majeur, en influençant la prise en charge de patients avec une atteinte de la conscience.

ABBREVIATIONS

AE	Adverse Event
AED	Antiepileptic drug
ASR	Annual Safety Report
CCI	Charlson Comorbidity Index
CEC	Competent Ethics Committee
cEEG	Continous video-electroencephalography
CER-VD	Commission cantonale d'éthique de la recherche sur l'être humain du canton de Vaud
CHUV	Centre Hospitalier Universitaire Vaudois
CPC	Cerebral Performance Categories
CRC	Centre de recherche clinique
CRF	Case Report Form
CTU	Clinical Trial Unit
ClinO	Ordinance on Clinical Trials in Human Research
eCRF	Electronic Case Report Form
EDC	Electronic Data Capture
EEG	Electroencephalography (*Electroencéphalogramme in French*)
FOPH	Federal Office of Public Health
FPFV	First Patient First Visit
GCP	Good Clinical Practice
HRA	Federal Act on Research involving Human Beings
HUG	Hôpitaux Universitaires de Genève
HVS	Hôpital du Valais
ICH	International Conference on Harmonization
ICF	Informed Consent Form
ICU	Intensive Care Unit(s)
IIT	Investigator-initiated Trial
ISF	Investigator Site File
ITT	Intention to treat
LPLV	Last Patient Last Visit
LRH	Loi fédérale relative à la recherche sur l'être humain
mRS	modified Rankin Scale
PI	Principal Investigator
rEEG	Routine video-electroencephalography
SDV	Source Data Verification
SE	*Status Epilepticus*
SOP	Standard Operating Procedure
SCTO	Swiss Clinical Trial Organization
TMF	Trial Master File
UNIL	University of Lausanne

STUDY SCHEDULE

Study period	Screening / Inclusion	Intervention period	Follow-up			
Days/Weeks/Months<br><br>Time (hours)	Day 1<br><br>T-5 - T0	Day 1-Day 2/3<br><br>T0 - T48	Day 3/4<br><br>T48 - T60	Day 7<br><br>-	Week 4 (Day 29)<br>-	Month 6 (Day 180)<br>-
**Visit Window**	***None***	***None***	**+ 24h**	**± 3 days**	**± 4 days**	**± 10 days**
**Assessments**						
Demographics	x					
Admission details (time, reason, hospital service)	x					
Glasgow Coma Scale or FOUR score	x					
Brain function alteration requiring EEG (Date, time and type)	x					
Previous seizures	x					
Informed consent	x	x	x	x	x	x
Eligibility check and inclusion	x					
Estimated body weight	x					
Charlson Comorbidity Index (CCI)	x					
Modified Ranking Scale (mRS)<br>**extrapolated before current hospitalization*	x*				x	x
SAPS II (only if available)	x					
Laboratory (only if available)	x					
Randomization	x					
EEG(s) details (Dates/times, electrodes numbers, use of interpretation algorithms)		x				
Detection of seizures (time, clinical correlate) and/or SE (time, type, STRESS score)		x				
Interictal epileptiform features (after ACNS)		x				
EEGs description		x				
Medication during EEG (except fluids, vitamins and feeding)		x				
SAEs potentially related to EEGs		x	x			
Changes in clinical management (treatment or new imaging)			x			
Last brain imaging results between 1 week before and 1 week after randomization					x	
Cerebral Performance Categories (CPC)					x	x
In-hospital infection requiring antibiotics					x	
Use of EEG after intervention					x	
Need of mechanical ventilation					x	
Discharge details (date, destination, back to work/school)					x	x

1. STUDY ADMINISTRATIVE STRUCTURE

1.1 Sponsor, Sponsor-Investigator

This clinical trial is an investigator-initiated clinical trial.

The sponsor is the Centre Hospitalier Universitaire Vaudois (CHUV), represented by the coordinating investigator:

Prof Andrea Rossetti
Service de neurologie
Rue du Bugnon 46, BH07
1011 Lausanne
Tel : 0041.21.314.11.90
Mobile : 0041.79.556.84.15
Fax : 0041.21.314.12.90
andrea.rossetti@chuv.ch

1.2 Principal Investigator(s)

- Site CHUV, Lausanne (coordinating site):

Prof Andrea Rossetti
Service de neurologie
Rue du Bugnon 46, BH07
1011 Lausanne
Tel : 0041.21.314.11.90
Mobile : 0041.79.556.84.15
Fax : 0041.21.314.12.90
andrea.rossetti@chuv.ch

- Site Hôpital du Valais, Sion:

Dr Vincent Alvarez
Service de neurologie
Hôpital du Valais (HVS) – Centre Hospitalier du Valais Romand
Hôpital de Sion
Avenue du Grand-Champsec 80
1951 Sion
Tel: 0041.27.603.86.59
Fax: 0041.27.603.44.38
Vincent.Alvarez@hopitalvs.ch

- Site Universitätsspital Basel, Basel:

Pr Stephan Rueegg
Head EEG, Epileptology and Neurointensive Care
Department of Neurology
University Hospital Basel
Petergraben 4
4031 Basel
Tel: 0041.61.265.47.57
Mobile: 0041.77.499.57.39

Fax: 0041.61.265.56.38
Stephan.Rueegg@usb.ch

- Site Inselspital, Bern:
  Pr Kaspar A. Schindler
  Director Sleep-Wake-Epilepsy Center
  University Clinic of Neurology
  Inselspital
  3010 Bern
  Tel: 0041.31.632.30.54
  Mobile: 0041.79.382.41.26
  Kaspar.Schindler@insel.ch

- Site Hôpitaux Universitaires de Genève (HUG), Genève:
  Pr Margitta Seeck
  Département de Neurologie
  Rue Gabrielle Perret-Gentil 4
  1205 Genève
  Tel: 0041.22.372.84.76
  margitta.seeck@hcuge.ch

1.3 Statistician ("Biostatistician")

PD Dr Raoul C. Sutter
University Hospital Basel
Medical Intensive Care Units ICU / CCU
Petergraben 4
4031 Basel
Tel: 0041.61.328.79.28
Mobile: 0041.78.838.85.99
Raoul.Sutter@usb.ch

1.4 Laboratory

Not applicable as no study-specific laboratory analyses will be performed.

1.5 Monitoring institution

The monitoring activities will be performed by the Lausanne Clinical Trial Unit (*Centre de recherche Clinique de Lausanne*) under the supervision of:

Prof Marc Froissart, CTU Director
Département de Formation et Recherche, CHUV / UNIL,
Mont Paisible 14
1011 Lausanne
Tel : 0041.21.314.61.84
marc.froissart@chuv.ch

1.6 Data Safety Monitoring Committee

In view of the study low risk, as continuous EEG and routine EEG are part of standard clinical care and will be performed according to clinical standards, no specific data safety monitoring committee will be constituted.

1.7 Any other relevant Committee, Person, Organisation, Institution

Trial management and data management

The trial management and the data management will be performed by the Lausanne Clinical Trial Unit (*Centre de recherche Clinique de Lausanne*) under the supervision of:

Prof Marc Froissart, CTU Director

Département de Formation et Recherche, CHUV / UNIL,

Mont Paisible 14

1011 Lausanne

Tel : 0041.21.314.61.84

marc.froissart@chuv.ch

2. ETHICAL AND REGULATORY ASPECTS

Before the study will be conducted, the protocol, the proposed information and consent forms as well as other study-specific documents will be submitted to a properly constituted Competent Ethics Committee (CEC) in agreement with local legal requirements, for formal approval. Any amendment to the protocol must as well be approved (if legally required) by these institutions.

The decision of the CEC concerning the conduct of the study will be made in writing to the Sponsor-Investigator before commencement of this study. The clinical study can only begin once approval from all required authorities has been received. Any additional requirements imposed by the authorities shall be implemented.

2.1 Study registration

Once approved by the CEC and before recruitment start, the study will be registered on www.clinicaltrial.gov registry and in addition, registered in a national language in the Swiss Federal Complementary Database (FOPH Portal).

2.2 Categorisation of study

This study is classified as risk category A as 1) the health-related intervention investigated entails only minimal risks and burdens and 2) the intervention under investigation is recommended by the European Society of Intensive Care Medicine and the American Clinical Neurophysiology Society for most patients with consciousness disorders.

2.3 Competent Ethics Committee (CEC)

This multicenter study will be submitted for approval by the sponsor-coordinating investigator to the lead CEC (i.e. Commission cantonale d'éthique de la recherche sur l'être humain du canton de Vaud, CER-VD). The local principal investigator at each site will ensure that approval from the appropriate local CEC is sought for the clinical study before recruitment start, however all local documents will be submitted by the sponsor-coordinating investigator in collaboration with the local principal investigator through the lead CEC.

All changes in the research activity will be reported to the lead CEC as per ClinO Art 34. If immediate safety and protective measures have to be taken during the conduct of the trial, the local principal investigator will communicate these measures to the coordinating investigator who will notify the lead CEC of these measures, and of the circumstances necessitating them, within 7 days (ClinO Art. 37, al.1). All serious adverse events occurring in participants that cannot be excluded to be attributable to the intervention under investigation will be reported by the coordinating investigator to the lead CEC within 15 days (ClinO Art 63). An annual safety report will also be submitted once a year to lead CEC by the coordinating investigator (ClinO Art. 43, al.1).

Premature study end or interruption of the study at one or several sites will be reported within 15 days by the sponsor-coordinating investigator to the lead CEC. The regular end of the study will be reported to the lead CEC within 90 days, the final study report will be submitted within one year after study end. Amendments will be reported according to chapter 2.10.

2.4 Competent Authorities (CA)

Not applicable.

2.5 Ethical Conduct of the Study

The study will be carried out in accordance to the protocol and with principles enunciated in the current version of the Declaration of Helsinki, the guidelines of Good Clinical Practice (GCP) issued by ICH and the Swiss Law. The lead CEC will receive annual safety and interim reports and be informed about study stop/end in agreement with local requirements.

2.6 Declaration of interest

The coordinating investigator, the trial statistician and the 4 local principal investigators are declaring to have no conflicts of interest within the context of this clinical trial.

2.7 Patient Information and Informed Consent

This study involves patients in an emergency situation. At the time of inclusion in the trial, all patients will not be able to give informed consent regarding their participation. Consequently, the following procedure regarding consent collection has to be strictly followed:

1. When patients are unconscious or considered by the investigator clinically unable to provide informed consent, they may be enrolled under the provisions in Article 30 (Research Projects in Emergency Situations) of the Human Research Act, but they should provide their own informed consent for continuing to participate in the study as soon as possible.

   In that case, the investigator will:

- Ensure that the patient has not expressed his right to object to participation in the study in any identifiable manner, including obtaining information orally from any available relative(s) on the patient’s will. If no relatives are accessible or available within the planned screening/enrolment time, the inclusion of the patient shall not be delayed and the wishes of the participant may be elucidated later, as soon as possible. This information will be clearly documented in the patient's medical files.
- In the event that a patient presents signs and symptoms showing unwillingness to participate in the study, the participant will be excluded from participation.
- Ensure that a physician who is not involved in the study and who safeguards the participant interests provides a written authorisation to enrol the patient. This physician will be a member of the emergency unit team, or another part of the intensive care facility, or another part of the neurology department. This physician has to be available within the screening and enrolment phase. By dating and signing a study-specific form, the independent physician confirms the protection of the patient’s interests as well as the guarantee of his/her medical follow-up. This site-specific CEC-approved form has to be signed by the independent physician and by the investigator before any study-specific intervention is made. The signed form will be retained as part of the study records.
- Ensure that informed consent for continuing to participate in the study is obtained post hoc from the patient as soon as possible, following the process described below.

2. When patients are capable of providing post-hoc informed consent (when the investigator is judging the patient to be able to consent), the investigator will:

- Explain to each patient the nature of the study, its purpose, the procedures involved (already done and to be done in the next study visits), the expected duration, the potential risks and benefits and any discomfort it may entail. Each patient will be informed that the participation in the study is voluntary and that he/she may withdraw from the study at any time and that withdrawal of consent will not affect his/her subsequent medical assistance and treatment. The participant must be informed that his/her medical records may be examined by authorized individuals other than their treating physician.
- Provide the patient with a site-specific CEC-approved participant information sheet and a consent form describing the study and providing sufficient information for the patient to make an informed decision about his participation to the study.
- Ensures that enough time will be given to the patient to decide whether to give post hoc consent. The patient should read and consider the statement before signing and dating the informed consent form. The consent form must also be signed and dated by the investigator.
- Ensures that a copy of the signed document has been given to the patient and that a copy will be retained as part of the study records.

3. When patients lack recovering full capacity after inclusion in emergency situation:

- If a subject is permanently lacking capacity, and if no statement of wishes formulated in a state of capacity is available, a proxy consent must be obtained from a person authorized to represent him/her (i.e. a person appointed in a patient decree or in an advance care directive; a deputy

with a right to act as representative in relation to medical procedures, or a next of kin (according to Art 378 of Swiss civil code).

- The consent will be requested as soon as possible but without excessive pressure, after being duly informed about the study. The patient's representative must confirm or invalidate the inclusion of the patient in the study based on the patient's presume wishes. The investigator will explain the nature of the study, its purpose, the procedures involved (already done and to be done in the next study visits), the expected duration, the potential risks and benefits and any discomfort it may entail. The representative will be provided a site-specific CEC-approved representative information sheet and a consent form describing the study and providing sufficient information for the representative to make an informed decision about the participation of the patient in the study. Enough time will be given to the representative to decide whether to give proxy consent. The representative should read and consider the statement before signing and dating the proxy informed consent form, and should be given a copy of the signed document. The consent form must also be signed and dated by the investigator and it will be retained as part of the study records.
- Assessment in time of patient's capacity to give informed consent will stop at the time of ICU/intermediate care unit discharge (mean stay time is around 10 days). In order to ensure that a patient's representative is still available on site before discharge, the consent of the representative will be requested at Day 7 (±3days).
  Of note, if a proxy consent is not obtainable as no representative is identified or cannot be reasonably contacted at that time, the patient will stay in the study and his/her data used in order not to compromise the results of the study.
- A re-evaluation of the participant's capacity to give informed consent will only be done at each study follow up "visits" by a study team member delegated at each site.

<u>Withdrawal of patients from the study and use of collected data in emergency situations:</u>

- If the patient refuses to give post hoc consent, he/she will be withdrawn from the study and the data collected so far will not be used for the study purposes.
- If the representative refuses to give proxy consent, the patient will be withdrawn from the study and the data collected so far will no longer be used for the study purposes.
- If a patient dies before it has been possible to obtain a consent or refusal from the representative and in the absence of a statement of wishes, his/her data collected up to the death will be used for study purposes (without oral consent from a next of kin or designated trusted person ; waiver to ClinO Art 16, alinea 2). This would prevent biasing the study validity (a mortality rate of about 30% is anticipated in view of the clinical situation of the recruited patients (see 11.2).

2.8 Participant privacy and confidentiality

The investigator affirms and upholds the principle of the participant's right to privacy and that they shall comply with applicable privacy laws. Especially, anonymity of the participants shall be guaranteed when presenting the data at scientific meetings or publishing them in scientific journals.

Individual subject medical information obtained as a result of this study is considered confidential and disclosure to third parties is prohibited. Subject confidentiality will be further ensured by utilising subject identification code numbers to correspond to treatment data in the computer files.

For data verification purposes, authorised representatives of the sponsor-coordinating investigator or an ethics committee may require direct access to parts of the medical records relevant to the study, including participants' medical history.

2.9 Early termination of the study

The sponsor-coordinating investigator may terminate the study prematurely according to certain circumstances, for example:

- insufficient participant recruitment,
- alterations in accepted clinical practice that make the continuation of a clinical trial unwise,
- early evidence of benefit or harm of the experimental intervention

2.10 Protocol amendments

Only the sponsor-coordinating investigator is allowed to amend the protocol. Local investigators may provide suggestions for a protocol amendment to the sponsor-coordinating investigator. Important protocol modifications will be submitted for approval to the lead CEC by the sponsor-coordinating investigator. Substantial amendments will only be implemented after approval of the CEC.

Under emergency circumstances, deviations from the protocol to protect the rights, safety and well-being of human subjects may proceed without prior approval of the sponsor-coordinating investigator and the CEC. Such deviations shall be documented and reported to the sponsor-coordinating investigator and the lead CEC as soon as possible.

All non-substantial amendments will be communicated to the lead CEC within the Annual Safety Report (ASR).

3. BACKGROUND AND RATIONALE

3.1 Background and Rationale

Electroencephalography (EEG) is a non-invasive tool to monitor the electrical correlates of brain function with a high temporal resolution. First described almost 90 years ago, it has experienced an impressive development in the last couple of decades following digitalization (software), the coupling with video recordings, and the exponential increase of hardware memory, allowing prolonged recordings and a straightforward and easy to apply application in clinical practice [1, 2]. In fact, it represents one of the most broadly used diagnostic tools in the neurology field: as an example, in 2015 at the CHUV, 2600 EEGs were performed, versus 1700 electro-neuro-myography (ENMG) and 1300 ultrasound (Doppler/Duplex) studies.

In the last decade, continuous video-EEG monitoring (cEEG), designating the EEG recording over several hours or days typically coupled with concomitant video recording and algorithms allowing quick interpretation of compressed recording times, is increasingly used in the intensive care units (ICUs), especially in North America [3-5]. The ICUs represent an environment related to considerable potential morbidity and mortality due to underlying critical diseases, not only involving primarily the brain, such as severe brain trauma, intracranial hemorrhage, subarachnoid hemorrhage, or ischemic stroke, but also in case of secondary brain injury, for example in patients with post-cardiac arrest encephalopathy, delirium, or sepsis-related encephalopathy [2, 6, 7]. The role of cEEG mostly resides in identifying seizures and status epilepticus (SE; i.e., prolonged seizures), which often do not show specific clinical correlates in this particular clinical setting [8] and may induce secondary brain injury [9], or to monitor treatment in patients with SE requiring general anesthesia for treatment [10]. Furthermore, it is also possible to detect changes in the cerebral electrical activity heralding blood flow variation, for example with vasospasms following subarachnoid hemorrhage [11, 12].

The American Clinical Neurophysiology Society has updated its recommendation for reporting particular EEG features in this setting, such as periodic and rhythmic patterns [13], contributing to a uniform nomenclature that should improve generalizability and inter-institutional comparability; they have been recently validated [14, 15].

In the last three years, the European Society of Intensive Care Medicine (ESICM) [6] and the American Clinical Neurophysiology Society [2, 7] published consensus statements intended to be guidelines regarding the use of cEEG (including, as a mandatory tool, video-correlation) in ICUs. cEEG video-monitoring is recommended for most patients with altered consciousness, in order to assist SE treatment management, especially in forms requiring pharmacological coma, and to rule out nonconvulsive seizures in brain-injured, or comatose patients with unexplained and altered consciousness. The North American guidelines further suggest its use in subjects with reduced level of consciousness under sedation or pharmacologically induced coma. cEEG should be recorded at least for 24 hours and interpreted at least twice daily [7]. However, especially in the European publication, the authors recognize that the supporting evidence is generally low, and that additional data are clearly necessary [6].

Some years ago, it has been shown that cEEG influences clinical practice inducing changes in patients' treatments [16], and recent evidence suggests that the patients' amount of time spent in the ICU having electrographic seizures, in other words the seizure "load", correlates with prognosis both in children [17] and adults [18]. However, no study has yet determined whether this finding simply reflects a more severe brain damage / dysfunction by the underlying etiology, or it is directly and causally related to outcome. In particular, to the best of our knowledge, it remains unclear if cEEG leads to a better prognosis in specific patients' populations; this represents a central unanswered question for clinical purposes. To date, only one population-based study relying on US discharge diagnoses, and therefore probably challenged by a considerable imprecision, has suggested that cEEG does improve outcome, with an odds ratio of 0.63 for in-hospital mortality (95% confidence interval; 0.51-0.76; $p < 0.001$) [3].

On the one hand, cEEG has a clearly superior sensitivity in uncovering seizures and SE as compared to routine spot video-EEG (rEEG, which typically lasts 20-30 minutes): a seminal retrospective study in an ICU-based population showed that the detection rate doubles from round 50% after one hour of recording to 95% after 48 hours, especially in comatose subjects [8]. Further evidence for the diagnostic yield of cEEG in patients with altered consciousness comes from a study revealing an increased detection rate of nonconvulsive SE after the introduction of cEEG as compared to historic controls with rEEG (monthly detection rates with cEEG 5.44 ±1.33; with rEEG 2.17 ±1.89, p=0.002) [19]. Nevertheless, cEEG is a time- and resource consuming procedure, requiring skilled technical personnel, regular assessments by electroencephalographers, and ideally the availability of dedicated, portable video-

EEG recording machines that may be connected to the hospital network for out-of-site inspection, as well as specially conceived supplies allowing emergent imaging procedures (e.g., computed tomography- and magnetic resonance imaging compatible electrodes [20]), namely overnight. Practical considerations have emerged in recent years. For example, it seems that a refined analysis of the first part of cEEG may allow stratifying the risk of encountering seizures in the subsequent recording: if no epileptiform discharge is detected in the first 30-120 minutes, the likelihood would be below 5% [21, 22]. Also, the use of automated software allowing the interpretation of compressed EEG seems to clearly improve efficacy in this setting, reducing the time required to analyze a cEEG by 78%, with minimal loss of sensitivity [23]. While large, mostly academic centers in North America have been using cEEG increasingly since the turning of the century, the vast majority of European hospitals - and all Swiss hospitals including university centers - still do not have the resources to apply cEEG to all patients with consciousness impairment in- or outside the ICUs, and therefore, disturbingly, are not complying with the current European guidelines [6].

There are also relevant financial considerations, which may act, at least in part, as an incentive to perform cEEG. Personal information we got in February 2016 from a colleague working in a hospital of the Harvard system (Boston, USA) illustrates that reimbursement through Medicare for a rEEG is $418.99, while for a cEEG is $2,184.31 per day; private insurances generate even higher amounts, but exact estimations depend on the company. Moreover, some centers bill additional costs for digital analysis.

It is surprising that despite the major interest in cEEG the only study supporting its usefulness in terms of prognosis is the aforementioned population-based assessment. Furthermore, there has been no attempt to compare cEEG with repeated rEEG. Given the preceding considerations, it seems very reasonable to consider that at the present time there is equipoise between cEEG and rEEG in terms of prognosis, and a controlled study appears urgently warranted.

Additionally, the role of quantitative EEG analysis in this clinical setting has received very scarce attention to date. An early study [24] tested if cEEG could help to differentiate between delirium, dementia and delirium coexistent with dementia, and the authors found that specific EEG variables were indeed helpful. However the EEG measures they applied only consisted of univariate linear approaches; also more recent studies in patients with acutely impaired consciousness [25] do not apply the modern powerful tools of network analysis, which appears much better suited to assess the delicate balance between functional integration and segregation in neuronal networks [26] that is currently thought to be the physiological basis for unimpaired consciousness [27]. We therefore expect that including network analysis in the study of cEEG will help to yield important diagnostic and prognostic information, which will be complementary to classical visual EEG analysis by experts. Furthermore, neural network analysis may also shed light onto the pathophysiology of impaired consciousness.

3.2 Investigational Product (treatment, device) and Indication

Continuous and routine video-EEG will be performed with recording machines normally used in clinical practice in the participating hospitals; for information, EEG is used in Swiss hospitals since the 1940’s, and continuous EEG since at least 15-20 years; video-EEG are routinely used since more than 10 years. To offer an idea of its frequency, there are between 1500-4000 yearly EEG studies in each participating hospital.

EEG recording and interpretation machines by different manufacturers will be used: Lausanne, Bern, Basel and Sion rely on the Nicolet system (Viasys, Neurocare, Madison, WI), while Geneva uses Micromed devices (Mogliano Veneto, TV, Italy). Persyst, Version 13 (San Diego CA), an automated EEG array display is used in the different participating centres (routinely in Lausanne and Sion).

Data for quantitative analyses will be exported in EDF+ format (readable with Matlab and other research softwares).

3.3 Preclinical Evidence

Not applicable

3.4 Clinical Evidence to Date

As mentioned above, only one US population-based study suggested that cEEG does improve outcome, with an odds ratio of 0.63 for in-hospital mortality (95% confidence interval; 0.51-0.76; p <

0.001) [3].

The use of cEEG in postanoxic patients was addressed recently by the team of the Sponsor-coordinating investigator [28]: in this particular diagnostic category the yield of cEEG seems comparable to that of a repeated rEEG [29]. In this study, in fact, the blinded review of 34 cEEG that were transformed post-hoc to 2 rEEG "clipped" each at the time of reactivity testing showed a comparable performance to that of the whole trace. Despite a relatively limited sample size, this represents a rationale to optimize the EEG use in resource-limited hospitals [30].

More recently, the same team retrospectively collected 29 consecutive patients with non-convulsive SE without coma, undergoing extended EEG; these were compared to an historical age-matched group of 58 patients managed with routine EEG only. While SE severity was similar in the two groups, with similar proportion of potential fatal etiologies (58% in the extended EEG group vs 60%, P=.529), and comparable acute hospitalization duration (median of 15 vs 11 days, P=.131), the extended EEG group received slightly more anti-epileptic drugs (median was three in both groups, P=.026). Distribution of the outcome categories at hospital discharge did not show any statistical difference (P=.129) (*Eskioglou et al., Acta Neurol Scand, in press*).

3.5 Dose Rationale / Medical Device: Rationale for the intended purpose in study (pre-market MD)

The time frame of 30-48 hours for cEEG and rEEG derives from the seminal study by Claassen et al, showing that within this time lapse the vast majority of seizures are detected in critical care patients[8].

3.6 Explanation for choice of comparator (or placebo)

Routine practice in the vast majority of European (and Swiss) hospitals, to date, is to perform rEEG. It is also common practice to repeat rEEG in several clinical situations [29]. Therefore, 2 rEEG were chosen as the (standard) comparator.

3.7 Risks / Benefits

Since the EEG is a noninvasive procedure, there are no significant risks that will be associated with either rEEG or cEEG. The only potentially challenging issue is a skin reaction under the electrodes in patients undergoing cEEG, which is extremely uncommon before seven to ten days of uninterrupted recording [1]. The participating centers, however, are currently already recording each year many cEEG in the proposed clinical setting, and skin reactions virtually never represent a problem for patients, and may be easily treated with local applications.

All files and records will be coded; therefore, there will be no risk of disseminating patients' identities. All study members will be submitted to strict confidentiality, according to current Swiss laws.

As a positive collateral effect of participating in the study, it is to expect that patients will tend to be followed up more carefully than in routine clinical practice.

There will be no blinding of the procedure to treating physicians and electroencephalographers. Such a blinding in this particular clinical setting would imply having two separate teams of clinical neurophysiologists in each center, which seems highly unpractical. Furthermore, withdrawing EEG information to the treating team would not only raise ethical questions, but also potentially impact on the clinical outcome at 6 months, therefore biasing the study. Importantly, in order to minimize confounding by EEG allocation (information bias), we foresee to assess at 6 month the primary and several secondary outcomes in a blinded fashion using a recently proposed structured interview for the functional outcome[31].

3.8 Justification of choice of study population

This study can by definition only be conducted in patients with altered mental state (see background), therefore not capable of judgment. It is in fact impossible to extrapolate findings from patients without altered mental state, as these in the vast majority of case do not need EEG for detection of seizures.

This study involves the use of EEG in an acute setting: it is not possible to postpone the procedure in order to await consent by the patient or legal representative if the latter is not readily available, as this is needed for clinical reasons.

The consent procedure in this emergency setting is already described under 2.7

4. STUDY OBJECTIVES

4.1 Overall Objective

The present study intends to assess if EEG signals, recorded continuously or intermittently, have a prognostic impact in patients with altered consciousness.

4.2 Primary Objective

The primary objective is to assess if cEEG offers advantages over rEEG in adult patients with altered consciousness in terms of survival at 6 months.

4.3 Secondary Objectives

The secondary objective is to assess if cEEG versus rEEG offers advantages in terms of functional outcome, seizure detection rate, hospital infections rate, need and duration of mechanical ventilation, length of stay and costs.

4.4 Safety Objectives

NA

5. STUDY OUTCOMES

5.1 Primary Outcome

- Mortality at 6 months (frequency).

5.2 Secondary Outcomes

- Functional outcome at four weeks and at six months (evaluated using the modified Rankin Scale (mRS), and the Cerebral Performance Categories (CPC) [32], ordinal),
- Evaluation at 4 weeks and 6 months of the ability to go back to work/school if previously working/at school (proportion),
- Seizure/SE detection rate, and time to detection after the start of EEG recording, and presence of concomitant clinical signs of seizures (proportion, resp. continuous variable)
- Detection of interictal potentially epileptiform features (spikes, spike and waves, sharp waves, isolated or repeated at <3Hz without any evolution; lateralized rhythmic delta activity [33] (proportion),
- Rate of in-hospital infections requiring antibiotic treatment at 4 weeks after first EEG (proportion),
- Need and duration of mechanical ventilation at 4 weeks after first EEG (proportion, resp. continuous variable),
- Duration if ICU and hospital stay (continuous variable),
- Patient destination after acute facility (home, rehab, nursing home, other; categorical)
- Change in clinical patient management (i.e., antiepileptic drugs (AED) introduced or stopped, AED increased or decreased, brain imaging procedure order) occurring during the 60 hours following the start of the first EEG (categorical).
- Correlation between quantitative EEG analysis and outcome.

5.3 Other Outcomes of Interest

- Global hospitalization costs intended as amount billed for each patient's acute hospital stay, assessed through the billing department of each hospital (continuous variable – stratified by site).

5.4 Safety Outcomes

NA

6. STUDY DESIGN

6.1 General study design and justification of design

This will be a multicenter randomized controlled trial assessing prognostic yield of cEEG. While there will be no blinding during the EEG procedure, as this would prove highly unpractical in the proposed setting; however, the most important outcomes will be assessed blindly (at 6 month). The study will be carried out in four university hospitals (of the five in Switzerland) and one large regional hospital, located both in the German- and the French-speaking part of the country. An RCT seems the best way to address the primary objective of this study, particularly in terms of minimization of biases.

It is expected to randomize 350 patients over a 2 year-recruiting period. Patients will receive either cEEG or 2 rEEG within the first 48 hours following randomization, and then will be followed up until 6 months after randomization.

6.2 Methods of minimising bias

A randomization stratified by center as well as a blinded assessment of the primary outcome at 6 month will be performed to minimize the bias.

6.2.1. Randomisation

Randomization (1:1) will be stratified by center and generated using the web-based secuTrial® software. Briefly, randomization by blocks of 4 (2 rEEG and 2 cEEG) will be set up in the system by the data managers of the CTU Lausanne. Using login and password, the local investigator (or his designee) will randomize patients after the eligibility check and just before the intervention by asking the system to provide him/her with a randomization number and intervention allocation. Randomization lists will be kept at the CTU Lausanne during the trial and transferred to the sponsor-coordinating investigator for interim analysis or at the end.

6.2.2. Blinding procedures

The intervention under investigation will not be blinded however the assessment of the primary outcome at 6 months will be performed by a study team member blinded to the intervention done in the study patients.

6.2.3. Other methods of minimising bias

The use of validated scales (modified Rankin Scale, Cerebral Performance Categories [32]) will also allow minimizing bias.

6.3 Unblinding Procedures (Code break)

Not applicable

7. STUDY POPULATION

7.1 Eligibility criteria

Inclusion criteria:

- In-patients aged ≥18 years, treated in an ICU or intermediate care unit
- Alteration of mental state of any etiology (i.e., primarily cerebral or not), with Glasgow-coma scale ≤11 or FOUR score ≤ 12.
- Need of an EEG to exclude seizures or SE, or to evaluate prognosis as per the treating physician or the consulting neurologist.
- Informed consent obtained for research in emergency situation according to Human Research Act (HRA) art 30-31 at the time of inclusion

Exclusion criteria:

- Clinical and/or electrographic *status epilepticus* < 96h before randomization
- Clinical and/or electrographic seizure < 36h before randomization
- Palliative care situation, in which detection of SE or seizures would not have any impact on the patient's care.
- High likelihood of needing a surgical intervention or an invasive diagnostic procedure within the next 48 hours according to the treating physician (as this would require cEEG removal).

7.2 Recruitment and screening

Three hundred and fifty (350) study patients will be recruited at the participating hospital through the normal clinical practice: all adults with altered mental state needing an EEG for clinical purposes, and not presenting any exclusion criterion, will be considered.

No recruitment can be made though outpatients or advertising, for instance.

7.3 Assignment to study groups

The study patients will be randomized by the local investigator (or his designee) after the eligibility check and just before the intervention using the web-based secuTrial® software. The system will provide the investigator with a randomization number and intervention allocation (rEEG or cEEG).

7.4 Criteria for withdrawal / discontinuation of participants

Patients who during the intervention period (30-48 hours) will be diagnosed with clinical or electrical seizures or SE (10-12% are expected (Alvarez et al, Clin Neurophysiol, in press)) will exit the intervention but stay in the study and be managed according to best clinical practice. A uniform definition of electrographic seizures (minimum time >10 seconds) and SE (minimum time >5 minutes) will be used: repetitive, rhythmic or periodic discharges or spike and wave patterns occurring at a frequency of <3 Hz together with evolution in frequency, location, or with electroclinical response to anticonvulsants; or occurring at a frequency of >3 Hz [8, 13, 34, 35]. These criteria, as well as interruption of the intervention as per the treating physician will not prevent assessment of the outcomes (only analyzed as "intention to monitor", according to the intervention group allocation).

Furthermore, refusal/withdrawal of consent from the patient or representative represents a mandatory discontinuation of the study.

8. STUDY INTERVENTION

8.1 Identity of Investigational Products (treatment / medical device)

No investigational products are investigated. This study aims to compare the prognostic yield of cEEG versus rEEG interventions.

Experimental Intervention

Patients randomized to cEEG will be recorded with at least 21 electrodes placed according to the international 10-20 system; occasionally, a reduced montage will be allowed in patients with extensive neurosurgical scars, according to good common practice. The electrodes type and the use of automated, quantitative EEG interpretation softwares will be at the discretion of the centers, but minimal technical requirements for EEG recording in patient with troubles of consciousness as declared by the International Federation of Clinical Neurophysiology have to be fulfilled [1]. Recordings will last a minimum of 30 and a maximum of 48 hours, in order to avoid definitive removal of electrodes during the night. During this time, one interruption to a maximum of two hours for diagnostic purposes (e.g., for neuroimaging) will be allowed. Reactivity testing using auditory and nociceptive stimuli will be performed at least twice during the recording time. Recordings will be visually interpreted by certified electroencephalographers (i.e., interpretation of the automated algorithm only won't be allowed) at least 3x during working days, and 2x during weekends and holidays, using the 2013 American Clinical neurophysiology nomenclature [13]; interpretations will be communicated within two hours of their completion to the treating team.

Control Intervention

Patients randomized to rEEG will be recorded with at least 21 electrodes placed according to the international 10-20 system; occasionally, a reduced montage will be allowed in patients with extensive neurosurgical scars, according to good common practice. Recordings will last between 20 and 30 minutes; two recordings will take place over a period of 24 to 48 hours. Reactivity testing using auditory and nociceptive stimuli will be performed once per recording. Recordings will be visually interpreted by certified electroencephalographers using the 2013 American Clinical neurophysiology nomenclature, as for the experimental intervention, and the interpretation will be communicated within two hours of its completion to the treating team (idem).

Packaging, Labelling and Supply (re-supply)

NA

Storage Conditions

NA

8.2 Administration of experimental and control interventions

Experimental Intervention

EEGs are routinely needed several times a day in each participating center for seizure detection in patients with altered consciousness. The time frame of 30-48 hours for cEEG and rEEG derives from the seminal study by Claassen et al, showing that within this time lapse the vast majority of seizures are detected in critical care patients[8]. Routine practice in the vast majority of European (and Swiss) hospital, to date, is to perform rEEG. It is also common practice to repeat rEEG in several clinical situations [29]. Therefore, 2 rEEG were chosen as the (standard) comparator.

EEGs are routinely used in all participating centres for clinical practice. EEG interpretation will be performed by certified study team members at each site, according to current standard of clinical care; for details please see 8.1.

Control Intervention

Please see above 8.1.

8.3 Dose / Device modifications

Patients that during the intervention period (30-48 hours) will be diagnosed with clinical or electrical seizures or SE (10-12% are expected (Alvarez et al, Clin Neurophysiol, in press)) will exit the intervention and managed according to best clinical practice. A uniform definition of electrographic seizures (minimum time >10 seconds) and SE (minimum time >5 minutes) will be used: repetitive,

rhythmic or periodic discharges or spike and wave patterns occurring at a frequency of <3 Hz together with evolution in frequency, location, or with electroclinical response to anticonvulsants; or occurring at a frequency of >3 Hz [8, 13, 34, 35]. Occurrence of these criteria, as well as interruption of the intervention as per the treating physician, will not prevent assessment of the primary outcome (intention to monitor).

8.4 Compliance with study intervention

NA

8.5 Data Collection and Follow-up for withdrawn participants

If participants or representative refuse to give consent, all data collected so far will not be used. However if participants or representative withdraw consent, all data collected so far will be used for the study purposes in order not to compromise the study.

If the intervention EEG is interrupted (please see 8.3), participants will stay in the study and data will be collected at all study time points as planned but analyzed in intention to monitor.

8.6 Trial specific preventive measures

There are no preventive measures. If seizures or status epilepticus will be detected during the intervention period, the treating clinicians will manage this according to standard care.

8.7 Concomitant Interventions (treatments)

There is no restriction at all for concomitant interventions and treatment. The intervention EEG can be temporarily interrupted (in case of cEEG) to perform a diagnostic procedure, if this is acutely needed. Duration of the interruption will be reported.

8.8 Study Drug / Medical Device Accountability

NA

8.9 Return or Destruction of Study Drug / Medical Device

NA

9. STUDY ASSESSMENTS

9.1 Study flow charts

Study period	Screening / Inclusion	Intervention period	Follow-up			
Days/Weeks/Months<br><br>Time (hours)	Day 1<br><br>T-5 - T0	Day 1-Day 2/3<br><br>T0 - T48	Day 3/4<br><br>T48 - T60	Day 7<br><br>-	Week 4 (Day 29)<br>-	Month 6 (Day 180)<br>-
**Visit Window**	***None***	***None***	**+ 24h**	**± 3 days**	**± 4 days**	**± 10 days**
**Assessments**						
Demographics	x					
Admission details (time, reason, hospital service)	x					
Glasgow Coma Scale or FOUR score	x					
Brain function alteration requiring EEG (Date, time and type)	x					
Previous seizures	x					
Informed consent	x	x	x	x	x	x
Eligibility check and inclusion	x					
Estimated body weight	x					
Charlson Comorbidity Index (CCI)	x					
Modified Ranking Scale (mRS)<br>**extrapolated before current hospitalization*	x*				x	x
SAPS II (only if available)	x					
Laboratory (only if available)	x					
Randomization	x					
EEG(s) details (Dates/times, electrodes numbers, use of interpretation algorithms)		x				
Detection of seizures (time, clinical correlate) and/or SE (time, type, STRESS score)		x				
Interictal epileptiform features (after ACNS)		x				
EEGs description		x				
Medication during EEG (except fluids, vitamins and feeding)		x				
SAEs potentially related to EEGs		x	x			
Changes in clinical management (treatment or new imaging)			x			
Last brain imaging results between 1 week before and 1 week after randomization					x	
Cerebral Performance Categories (CPC)					x	x
In-hospital infection requiring antibiotics					x	
Use of EEG after intervention					x	
Need of mechanical ventilation					x	
Discharge details (date, destination, back to work/school)					x	x

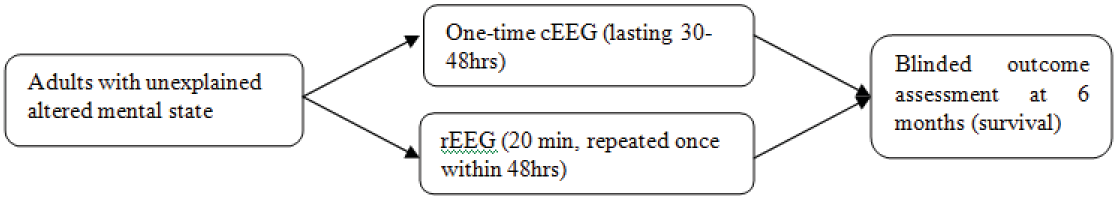

9.2 Assessments of outcomes

9.2.1. Assessment of primary outcome

Mortality at 6 months will be assessed through a structured phone interview (to the patient, representative or general practitioner, depending on the patient's medical condition, consent status and location) by a study team member blinded to the EEG intervention.

9.2.2. Assessment of secondary outcomes

The following secondary outcomes will be assessed. Of note, the assessments at 4 weeks will be done through chart review or by phone to the patient, representative or general practitioner, depending on the patient's medical condition, consent status and location. All secondary outcomes at 6 months will be assessed through a structured phone interview (to the patient, representative or general practitioner, depending on the patient's medical condition, consent status and location) by a study team member blinded to the EEG intervention.

- Functional outcome (modified Rankin Scale (mRS), Cerebral Performance Categories (CPC) [32], ordinal) , assessed at 4 weeks through chart review or phone and at 6 months through structured phone interview.
- Back to work/school if previously working/at school, assessed at 4 weeks through chart review or phone and at 6 months through structured phone interview.
- Seizure/SE detection rate, and time to detection after the start of EEG recording, and presence of concomitant clinical signs of seizures, assessed through chart review at 48hours.
- Detection of interictal potentially epileptiform features (spikes, spike and waves, sharp waves, isolated or repeated at <3Hz without any evolution; lateralized rhythmic delta activity [33] (proportion)), assessed through chart review at 48hours.
- Rate of in-hospital infections requiring antibiotic treatment assessed at 4 weeks through chart review.
- Need and duration of mechanical ventilation, assessed at 4 weeks through chart review.
- Duration if ICU and hospital stay, assessed at 4 weeks through chart review.
- Patient destination after acute facility, assessed at 4 weeks through chart review or phone and at 6 months through structured phone interview.
- Change in clinical patient management (i.e., AED introduced or stopped, AED increased or decreased, brain imaging procedure order) occurring during the 60 hours following the start of the first EEG (categorical), assessed through chart review at 48-60 hours.
- Correlation between quantitative EEG analysis and outcome assessed, assessed at the study end by the Bern team in a blinded fashion.

9.2.3. Assessment of other outcomes of interest

- Global hospitalization costs, intended as amount billed for each patient's acute hospital stay, assessed through the billing department of each hospital, assessed at study end (unblinded).

9.2.4. Assessment of safety outcomes

NA

9.2.5. Assessments in participants who prematurely stop the study

NA

9.3 Procedures at each visit

Study-specific site guidelines detailing screening, consent procedure and data collection for each

study period/timepoint will be prepared prior to study initiation.

Screening and enrolment study period – Day 1 (T-5 to T0)

This first study "visit" will be dedicated to screening and enrolment of the patient in the trial. Due to the emergency situation this visit will last 5 hours, at maximum. During this visit, evaluation of eligibility, baseline assessments and randomization will be performed. All information for the study outcomes evaluation will be collected from the medical charts.

Intervention study period – Day 1 to Day2/3 (T0 to T48)

During this study period, the intervention (cEEG or rEEG) will be performed. Concomitant medication (except fluids, vitamins and feeding) and SAE that cannot be excluded to be attributable to the intervention cEEG will also be recorded. All information for the study outcomes evaluation will be collected from the medical charts.

Follow up at Day 3/4, T48 to T60 hours

EEG-related findings and changes in clinical trial management will be collected from medical charts

Follow up at Week 4

Several outcome findings (see detailed study flow chart and 9.2) will be collected from medical chart if the patient is still hospitalized at site or by phone to the patient, representative or general practitioner, depending on the patient's medical condition, consent status and location.

Follow up at Month 6

Several outcome findings (see detailed study flow chart and 9.2) will be collected through a structured phone interview (to the patient, representative or general practitioner, depending on the patient's medical condition, consent status and location) by a study team member delegated by the principal investigator, blinded to the EEG intervention and centralized for all trial sites.

10. SAFETY

10.1 Interventions studies

As already stipulated under 3.7, since the EEG is a noninvasive procedure, there are no significant risks that will be associated with either rEEG or cEEG. The only potentially challenging issue is a skin reaction under the electrodes in patients undergoing cEEG, which is extremely uncommon before seven to ten days of uninterrupted recording [1]. The participating centers, however, are currently already recording each year many cEEG in the proposed clinical setting, and skin reactions virtually never represent a problem for patients, and may be easily treated with local applications.

10.1.1. Definition, assessment and reporting of (serious) adverse events and other safety related events

An Adverse Event (AE) is any untoward medical occurrence in a patient or a clinical investigation participant administered a pharmaceutical product and which does not necessarily have a causal relationship with the study procedure. An AE can therefore be any unfavourable and unintended sign (including an abnormal laboratory finding), symptom, or disease temporally associated with the use of a medicinal (investigational) product, whether or not related to the medicinal (investigational) product (ICH E6 1.2).

As per ClinO Art.63, a Serious Adverse event (SAE) is defined as any event that :

a. requires inpatient treatment not envisaged in the protocol or extends a current hospital stay;

b. results in permanent or significant incapacity or disability;

c. is life-threatening or results in death; or

d. causes a congenital anomaly or birth defect.

During the intervention phase of the study, all serious adverse events (SAEs) that cannot be excluded to be attributable to the intervention under investigation, will be collected, fully investigated and documented in source documents and case report forms (CRF). It is not expected to experience SAE related to the intervention occurring after the intervention.

Additionally, all these events will be reported by the local investigator to the sponsor-coordinating investigator as soon as possible (within 24h). The sponsor-coordinating investigator will report these events to the lead CEC within 15 days (ClinO Art. 63).

If immediate safety and protective measures have to be taken during the conduct of the clinical trial, the local investigator will inform the sponsor-coordinating investigator as soon as possible. The latter will notify the lead CEC of these measures, and of the circumstances necessitating them, within 7 days (ClinO Art.37).

Once a year, the sponsor-coordinating investigator will submit to the lead CEC an annual safety report summarising the safety of participants (ClinO Art.43).

10.1.2. Follow up of (Serious) Adverse Events

SAEs that cannot be excluded to be attributable to the intervention under investigation will be followed until resolution or stabilisation. Participants with ongoing SAEs at study termination (very improbable) will be further followed up until recovery or until stabilisation of the disease after termination.

11. STATISTICAL METHODS

11.1 Hypothesis

The null hypothesis is that cEEG, will not improve patients' survival (reduce mortality) at 6 months as compared to rEEG.

11.2 Determination of Sample Size

According to the only available study broadly addressing this issue [3], patients with consciousness disorders and cEEG have a 25% mortality (corresponding to 75% survival rate) and patients without cEEG a 39% mortality (implying a 61% survival rate). Considering a power of 0.8, an alpha error of 0.05, and applying a 2-sided test, 2x174 patients (348 in total) would be needed to detect a difference of 61% versus 75% in survival (χ^2 test for independent samples, Stata version 12, College station, TX). This represents a reasonable difference of non-debatable clinical relevance.

11.3 Statistical criteria of termination of trial

There are no stopping rules for individual participants. The discontinuation criteria for patients are the post hoc or proxy consent refusal/withdrawal, only. An interim analysis of the primary outcome will be performed after recruitment of the first 100 patients if the difference of 14% in survival at six months between the two arms will be met, the study will be interrupted.

11.4 Planned Analyses

Analysis of the outcomes

All analyses will be carried out by a professional statistician. At study completion, the two interventional groups will be compared regarding survival at six months as "intention to monitor" (predefined analysis for the primary endpoint) and "per protocol", adjusted for potential confounders (logistic regressions).

Analysis of each secondary endpoint will be also conducted using univariate and multivariate approaches.

For univariate approaches, frequency tables (χ^2 or Fisher exact tests, as needed), t-test, Mann-Whitney U, and Kaplan-Meier tests will be applied, as needed.

For the multivariate approaches, stepwise logistic regression models will be performed to identify independent outcome predictors among those found to have a p <0.1 on univariate analyses. Accordingly, risk-prediction models will be set-up and "Goodness-of-fit" of these models will be evaluated using a Hosmer-Lemeshow test to ensure the quality of calibration. Solid predictors will be used to predict survival and functional outcome at six months in a model. To evaluate the performance of these models, sensitivity, specificity, positive predictive value, negative predictive value, unweighted accuracy (using exact binomial 95% CI), area under the Receiver Operating Characteristic (ROC) curve, and reclassification analysis (Net Reclassification Improvement – NRI) will be calculated [36].

Quantitative EEG analysis

Modern concepts about the neurophysiological basis of unimpaired consciousness propose that a stable balance between functional segregation and integration has to be maintained [26, 37]. Deviations from this physiological state may cause impairment of consciousness and can be quantitatively assessed by studying functional networks derived from EEG signals [38] as previously done for example for peri-seizure recordings [39]. We will use methods of symbolic analysis [40] and information theory [41] to detect pathologic states of hypersegregation or hyperintegration, and to assess the redundancy of single signals and the synchrony between all the EEG signals, in order to detect states of pathologic hypersegregation or hyperintegration. Specifically, we will use the slope cross correlation [42] as a linear, and horizontal visibility graphs [43] as a non-linear measure to compute a weighted adjacency matrix consisting of all the pairwise interdependences between EEG signals. From these matrices, we will derive maximal spanning trees [38, 44], which are uniquely defined acyclic subgraphs that allow to efficiently characterize – by computing their diameter and so-called leafs - the integrative and segregative characteristics of the underlying functional brain networks. We will compare these modern measures of network topology to classical univariate characteristics, such as the relative delta power averaged across all EEG signals. All the necessary algorithms have already been programmed and tested in Matlab by one of the principal investigators (K.S.) and are ready for use.

Repetitive routine EEG will only provide sparse temporal information about the evolution of these

network characteristics. Continuous EEG on the other hand should allow to precisely monitor the speed, variability and consistency of these changes. We hypothesize that the faster and the more robust the above described EEG network measures will re-attain a balanced state of integration and segregation, the better the patients' outcome will be. These measures and their dynamics, which reflect neuronal activity, both on smaller and larger spatial scales, will then be correlated with clinical outcomes and tested for their predictive value through multivariate analysis approaches.

11.4.1. Datasets to be analysed, analysis populations

The patients data will be analyzed as "intention to monitor EEG", i.e.: forming two groups (one of patients allocated to cEEG, the other to those allocated to rEEG). A per-protocol analysis will also be provided for the primary outcome.

According to current experience with a registry of post-cardiac arrest patients (personal observation in CHUV, Lausanne), subjects lost to follow-up represent 1-2% of the cohort. Available data will be used for secondary outcomes, but subjects will be excluded from analysis of the primary outcome.

11.4.2. Primary Analysis

Analyses of the clinical outcomes at study completion will be performed by the team of PD Dr Raoul Sutter, University Hospital of Basel, delegated by the sponsor-coordinating investigator and using the clinical trial datasets provided by the data management team of the CRC Lausanne according to adequate format requested by the statistics.

11.4.3. Secondary Analyses

Quantitative EEG analyses at study completion will be performed by the team lead by Prof. K Schindler, Inselspital Bern, delegated by the sponsor-coordinating investigator and using the EEG datasets compiled by the same team based on information extracted from coded EEG traces.

11.4.4. Interim analyses

An interim analysis of the primary outcome will be performed after recruitment of the first 100 patients: if the difference of 14% in survival at six months between the two arms will be met, the study will be interrupted. This analysis will be conducted by the team of the sponsor-coordinating investigator in Lausanne and using the intermediate clinical trial datasets provided by the data management team of the CRC Lausanne.

11.4.5. Safety analysis

Not foreseen.

11.4.6. Deviation(s) from the original statistical plan

Any deviation from the planned analyses will be justified and reported in the intermediate and final clinical study report.

11.5 Handling of missing data and drop-outs

Lost to follow up and drop outs rate is estimated around 1-2%. Dropout patients are defined as : i) patients who refuse or withdraw consent to participate in the trial ii) patients whom representative refuse or withdraw consent, iii) randomized patients for whom the intervention did not start iv) Patients for whom new information regarding one of the eligibility criteria arise during intervention (i.e. delayed knowledge about previous epileptic seizures/SE or statement of wishes or need for invasive procedure).

Dropout patients will be replaced except those who withdraw consent /patients whom representative withdraw consent as partial analyzable data is available

12. QUALITY ASSURANCE AND CONTROL

12.1 Data handling and record keeping / archiving

12.1.1 Case Report Forms

All trial data of each patient will be recorded from the source documents in a secured electronic Case Report Form (eCRF, secuTrial® software) independently managed by the Clinical Trial Unit, Lausanne, warranting data integrity, security, quality and traceability. Only authorized study collaborators (delegated by the local investigator at each site) will be allowed to proceed to eCRF entries/modifications. eCRFs will be kept current to reflect patient status at each phase during the course of study. Participants must not be identified in the eCRF by name or initials and birth date. Appropriate coded identification must be used according to study-specific standard operating procedures elaborated by the sponsor-coordinating investigator.

Both EEG quantitative analysis and general hospitalization costs assessments will not be reported in the eCRF as these evaluations will be done at the end of the study and directly integrated in the clinical trial database following a data entry validation procedure.

12.1.2. Specification of source documents

Source data must be available at each site to document the existence of the study participants. Source data must include the original documents relating to the study. The electronic patient medical file will consist in the source data at all sites. The only exceptions will be:

- The dates/signatures of the Informed consent forms
- The randomization number and intervention allocation (attributed by the web-based randomization system following a randomization list)
- The SAE assessment (using a study-specific paper SAE form)
- The 6-month phone interview (data collected on a paper form – for blinding reasons).

All paper source data will be archived at site in the Investigator Site File (ISF).

EEG traces will be stored in the clinical EEG database of each center, as clinical information deriving from it will be readily accessible to treating clinicians, and as interpretations will be written as in clinical practice on the same day of recording. Routinely, after the interpretation of each recording, related videos will be deleted. In a small proportion of patients (<5%) it is general practice to save short video-clips of salient clinical events (some seconds to a few minutes in total) for subsequent clinical judgment, according to the clinical evolution. These files are separated from the original (now video-less) recording. If every video had to be deleted for this study, this would expose participating patients to a limitation regarding their clinical care, as compared to patients not participating at the study; we feel that this would be ethically unacceptable.

12.1.3. Record keeping / archiving

All study-specific data and documents related to a specific site will be archived at this site in a site-specific Investigator Site File (ISF) maintained up-to-date during the trial by the local investigator (or his designee) as per GCP. All study-specific documents will be archived at the sponsor site in a Trial Master File (TMF) maintained up-to-date during the trial by the sponsor team as per GCP.

All study-specific data and documents must be archived at site and at the sponsor's office for a minimum of 10 years after study termination or premature termination of the clinical trial.

12.2 Data management

12.2.1. Electronic Data Capture (EDC) system and underlying database

Trial data of each patient will be recorded from the source documents in an secured web-based interfaced eCRF (secuTrial® software) independently developed and managed by the Clinical Trial Unit, Lausanne under the Swiss Clinical Trial Organisation (SCTO) CTU-shared license, warranting data integrity, security, quality and traceability.

The database itself will be stored on secured servers under the responsibility of the CHUV IT Department beneficiating of the institution safety policies and secured environment.

12.2.2. Data entry, and validation process

Data will be entered in the eCRF by study collaborators delegated by the local investigator at each site and previously trained by the data management team.

Access to data will be granted to the local investigator or study collaborators explicitly allowed to access data by the local investigator. Every study collaborator granted for data entry will access the system through an individual login/password. Automated univariate alerts will be set to secure data at time of entry.

No one will be permitted to alter data in the eCRF, except the local investigator or his designee in case an error has been noted during monitoring or electronic validation. All data alteration will be automatically traced in the software (secuTrial®). When complete, each eCRF will be validated by the local investigator using dedicated entry fields. The built-in traceability of the eCRF software (secuTrial) will guarantee that this signature is valid (login/password, date and time of entry).

Backup of electronic data are built-in in the eCRF software (secuTrial®) and on CHUV servers.

EEG traces for quantitative analysis will be collected at each site, once a year, by an investigator from the Bern team (delegated by each local investigator). These traces (without video) will be coded on each clinical site, transformed to *European Data Format plus* (EDF+) format, and stored for 10 years in a secured space at the Inselspital Bern EEG unit.

12.2.3. Electronic and central data validation

Data validity, coherence, and completeness will be assessed at several steps.

- First, control rules will be implemented in the data entry software (secuTrial®).
- Second, coherence of study plan and data collection will be regularly assessed by the local investigator throughout the study.
- Then, monitoring performed by the Clinical Trial Unit, Lausanne (see below) will encompass partial source data verification and CRF completeness check.
- Finally, coherence and completeness will be checked by data management to assess data completeness, data consistency, generating automated and manual queries to ensure data cleaning, data reconciliation, and medical data coding (events, safety data, medications…) prior to database-lock.

The clinical trial database constituted from EEG quantitative data will be validated using a double entry validation system.

12.2.4. Analysis and archiving

After study database-lock, all data will be extracted into an EDC exported database from which will be derived several clinical trial datasets (all together forming the clinical trial analysis database) upon format requested by the statisticians.

The study database (including traceability metadata) and the clinical trial analysis database will be stored for ten years on electronic folders secured on the CHUV servers and protected by passwords. Access to the datasets will be granted by the sponsor-coordinating investigator to collaborators delegated for statistical analysis. To ensure long-tem storage durableness, standard file formats (CSV, HDF5…) will be chosen.

Data extracted from the EEG traces for quantitative analysis will be entered in a separate clinical trial EEG database and analyzed in Matlab (Mathworks,Natick, USA). The files will be stored for ten years on secured servers protected by passwords at Inselspital Bern EEG unit, ready for statistical analysis performed by the same team.

12.2.5. Data sharing plan

In order to comply with ICMJE data sharing requirements for clinical trials [45], a data sharing plan will be developed, identifying a data repository that will be entrusted with storing, curating and sharing anonymized data used for analyses presented in study publications. A governance of access to data will be defined accordingly.

12.3 Monitoring

Monitoring will be performed according to ICH Good Clinical Practice (GCP) by the Clinical Trial Unit, Lausanne. A monitor (not implicated in the trial management) will perform monitoring following a risk-adapted monitoring plan and written Standard Operating Procedures (SOPs). The monitor will verify that the clinical trial is conducted and data are generated, documented and reported in compliance with the protocol, GCP and the applicable regulatory requirements. Basically, a site initiation visit,

several interim monitoring visits and a site closure visit will be organised by the monitor at each clinical site. On-site and remote (checking the eCRF) monitoring will be performed in parallel. The local investigator will provide direct access to all trial related source data/documents and reports for the purpose of monitoring and will answer monitors’ questions during monitoring visits.

12.4 Audits and Inspections

No trial audit is planned. However in case of an audit by the sponsor and/or inspection by the CEC, the study documentation and source data/documents will be accessible to auditors/inspectors and questions will be answered during audits/inspections. All involved parties will keep the participant data strictly confidential.

12.5 Confidentiality, Data Protection

Data protection and confidentiality will be guaranteed. Direct access to source documents will be permitted for purposes of monitoring (12.3), audits and inspections (12.4).

The sponsor-coordinating investigator and statisticians will have access to protocol, datasets and statistical code during and after the study. All local investigators (and delegated study collaborators) will have access to all study documents (protocol, procedures, source documents and eCRF) during the study.

12.6 Storage of biological material and related health data

No study-specific biological material will be sampled during this study.

13. PUBLICATION AND DISSEMINATION POLICY

After study completion, the results of the present study will be communicated using abstracts in national and international congresses. Scientific papers will be written by all the study team (i.e the sponsor-coordinating investigator and/or the local investigators) and submitted to peer-reviewed scientific journals. All authors will have to make a substantial intellectual contribution to the paper, including at least a detailed critical revision. No medical writer is foreseen.

14. FUNDING AND SUPPORT

14.1 Funding

This clinical trial has been granted by the Swiss National Science Foundation (SNSF grant 320030_169379).

14.2 Other Support

None

15. INSURANCE

With regard to potential damages which participants may suffer as a result of the study, the CHUV takes the responsibility as the study sponsor in accordance with the applicable legal provisions.

16. REFERENCES

1. Alvarez V, Rossetti AO. Clinical Use of EEG in the ICU: Technical Setting. Journal of clinical neurophysiology : official publication of the American Electroencephalographic Society 2015;32:481-485.

2. Herman ST, Abend NS, Bleck TP, et al. Consensus statement on continuous EEG in critically ill adults and children, part II: personnel, technical specifications, and clinical practice. Journal of clinical neurophysiology : official publication of the American Electroencephalographic Society 2015;32:96-108.

3. Ney JP, van der Goes DN, Nuwer MR, Nelson L, Eccher MA. Continuous and routine EEG in intensive care: utilization and outcomes, United States 2005-2009. Neurology 2013;81:2002-2008.

4. Friedman D, Claassen J, Hirsch LJ. Continuous electroencephalogram monitoring in the intensive care unit. Anesth Analg 2009;109:506-523.

5. Gavvala J, Abend N, LaRoche S, et al. Continuous EEG monitoring: a survey of neurophysiologists and neurointensivists. Epilepsia 2014;55:1864-1871.

6. Claassen J, Taccone FS, Horn P, et al. Recommendations on the use of EEG monitoring in critically ill patients: consensus statement from the neurointensive care section of the ESICM. Intensive care medicine 2013;39:1337-1351.

7. Herman ST, Abend NS, Bleck TP, et al. Consensus statement on continuous EEG in critically ill adults and children, part I: indications. Journal of clinical neurophysiology : official publication of the American Electroencephalographic Society 2015;32:87-95.

8. Claassen J, Mayer SA, Kowalski RG, Emerson RG, Hirsch LJ. Detection of electrographic seizures with continuous EEG monitoring in critically ill patients. Neurology 2004;62:1743-1748.

9. Vespa P, Tubi M, Claassen J, et al. Metabolic Crisis occurs with Seizures and Periodic Discharges after Brain Trauma. Annals of neurology 2016.

10. Rossetti AO, Lowenstein DH. Management of refractory status epilepticus in adults: still more questions than answers. The Lancet Neurology 2011;10:922-930.

11. Gollwitzer S, Groemer T, Rampp S, et al. Early prediction of delayed cerebral ischemia in subarachnoid hemorrhage based on quantitative EEG: A prospective study in adults. Clinical neurophysiology : official journal of the International Federation of Clinical Neurophysiology 2015;126:1514-1523.

12. Claassen J, Perotte A, Albers D, et al. Nonconvulsive seizures after subarachnoid hemorrhage: Multimodal detection and outcomes. Annals of neurology 2013.

13. Hirsch LJ, LaRoche SM, Gaspard N, et al. American Clinical Neurophysiology Society's Standardized Critical Care EEG Terminology: 2012 version. Journal of clinical neurophysiology : official publication of the American Electroencephalographic Society 2013;30:1-27.

14. Gaspard N, Hirsch LJ, LaRoche SM, Hahn CD, Westover MB, Critical Care EEGMRC. Interrater agreement for Critical Care EEG Terminology. Epilepsia 2014;55:1366-1373.

15. Westhall E, Rosen I, Rossetti AO, et al. Interrater variability of EEG interpretation in comatose cardiac arrest patients. Clinical neurophysiology : official journal of the International Federation of Clinical Neurophysiology 2015;126:2397-2404.

16. Kilbride RD, Costello DJ, Chiappa KH. How seizure detection by continuous electroencephalographic monitoring affects the prescribing of antiepileptic medications. Arch Neurol 2009;66:723-728.

17. Payne ET, Zhao XY, Frndova H, et al. Seizure burden is independently associated with short term outcome in critically ill children. Brain : a journal of neurology 2014;137:1429-1438.

18. De Marchis GM, Pugin D, Meyers E, et al. Seizure burden in subarachnoid hemorrhage associated with functional and cognitive outcome. Neurology 2016;86:253-260.

19. Sutter R, Fuhr P, Grize L, Marsch S, Ruegg S. Continuous video-EEG monitoring increases detection rate of nonconvulsive status epilepticus in the ICU. Epilepsia 2011;52:453-457.

20. Vulliemoz S, Perrig S, Pellise D, et al. Imaging compatible electrodes for continuous electroencephalogram monitoring in the intensive care unit. Journal of clinical neurophysiology : official publication of the American Electroencephalographic Society 2009;26:236-243.

21. Shafi MM, Westover MB, Cole AJ, Kilbride RD, Hoch DB, Cash SS. Absence of early epileptiform abnormalities predicts lack of seizures on continuous EEG. Neurology 2012;79:1796-1801.

22. Westover MB, Shafi MM, Bianchi MT, et al. The probability of seizures during EEG monitoring in critically ill adults. Clinical neurophysiology : official journal of the International Federation of Clinical Neurophysiology 2015;126:463-471.

23. Moura LM, Shafi MM, Ng M, et al. Spectrogram screening of adult EEGs is sensitive and efficient. Neurology 2014;83:56-64.

24. Jacobson SA, Leuchter AF, Walter DO. Conventional and quantitative EEG in the diagnosis of delirium among the elderly. J Neurol Neurosurg Psychiatry 1993;56:153-158.

25. Thomas C, Hestermann U, Kopitz J, et al. Serum anticholinergic activity and cerebral cholinergic dysfunction: an EEG study in frail elderly with and without delirium. BMC neuroscience 2008;9:86.

26. Sporns O. Network attributes for segregation and integration in the human brain. Current opinion in neurobiology 2013;23:162-171.

27. Oizumi M, Albantakis L, Tononi G. From the phenomenology to the mechanisms of consciousness: Integrated Information Theory 3.0. PLoS computational biology 2014;10:e1003588.

28. Rossetti AO, Urbano LA, Delodder F, Kaplan PW, Oddo M. Prognostic value of continuous EEG monitoring during therapeutic hypothermia after cardiac arrest. Crit Care 2010;14:R173.

29. Alvarez V, Sierra-Marcos A, Oddo M, Rossetti AO. Yield of intermittent versus continuous EEG in comatose survivors of cardiac arrest treated with hypothermia. Crit Care 2013;17:R190.

30. Rossetti AO, Rabinstein A, Oddo M. Neurological prognostication of comatose patients after cardiac arrest: shedding light into the dark Lancet Neurology 2016;in press.

31. Mak M, Moulaert VR, Pijls RW, Verbunt JA. Measuring outcome after cardiac arrest: construct validity of Cerebral Performance Category. Resuscitation 2016;100:6-10.

32. Booth CM, Boone RH, Tomlinson G, Detsky AS. Is this patient dead, vegetative, or severely neurologically impaired? Assessing outcome for comatose survivors of cardiac arrest. Jama 2004;291:870-879.

33. Gaspard N, Manganas L, Rampal N, Petroff OA, Hirsch LJ. Similarity of Lateralized Rhythmic Delta Activity to Periodic Lateralized Epileptiform Discharges in Critically Ill Patients. JAMA neurology 2013.

34. Beniczky S, Hirsch LJ, Kaplan PW, et al. Unified EEG terminology and criteria for nonconvulsive status epilepticus. Epilepsia 2013;54 Suppl 6:28-29.

35. Sutter R, Kaplan PW. The neurophysiologic types of nonconvulsive status epilepticus: EEG patterns of different phenotypes. Epilepsia 2013;54 Suppl 6:23-27.

36. Steyerberg EW, Vickers AJ, Cook NR, et al. Assessing the performance of prediction models: a framework for traditional and novel measures. Epidemiology 2010;21:128-138.

37. Tononi G, Koch C. Consciousness: here, there and everywhere? Philosophical transactions of the Royal Society of London Series B, Biological sciences 2015;370.

38. Stam CJ, Tewarie P, Van Dellen E, van Straaten EC, Hillebrand A, Van Mieghem P. The trees and the forest: Characterization of complex brain networks with minimum spanning trees. International journal of psychophysiology : official journal of the International Organization of Psychophysiology 2014;92:129-138.

39. Schindler K, Gast H, Goodfellow M, Rummel C. On seeing the trees and the forest: single-signal and multisignal analysis of periictal intracranial EEG. Epilepsia 2012;53:1658-1668.

40. Daw C, Finney C, Tracy E. A review of symbolic analysis of experimental data. Rev Sci Instrum 2003;74:915-920.

41. Luque B, Lacasa L, Ballesteros F, Luque J. Horizontal visibility graphs: exact results for random time series. Physical review E, Statistical, nonlinear, and soft matter physics 2009;80:046103.

42. Rummel C, Goodfellow M, Gast H, et al. A systems-level approach to human epileptic seizures. Neuroinformatics 2013;11:159-173.

43. Lacasa L, Nicosia V, Latora V. Network structure of multivariate time series. Scientific reports 2015;5:15508.

44. van Diessen E, Otte WM, Braun KP, Stam CJ, Jansen FE. Does sleep deprivation alter functional EEG networks in children with focal epilepsy? Frontiers in systems neuroscience 2014;8:67.

45. Taichman DB, Backus J, Baethge C, et al. Sharing clinical trial data: a proposal from the International Committee of Medical Journal Editors. Rev Med Chil 2016;144:11-13.

17. APPENDICES

NA
