# Supplementary material for: Continuous vs Routine Electroencephalogram in Critically Ill Adults With Altered Consciousness and No Recent Seizure: A Multicenter Randomized Clinical Trial
Source: JAMA Neurol. 2020 Jul 27;77(10):1–8. doi: 10.1001/jamaneurol.2020.2264 (PMC7385681; doi:10.1001/jamaneurol.2020.2264)
Supplement: Supplement 2. — eTable 1. Exploratory analysis of primary outcome (mortality at 6 months) and functional outcomes eFigure 1. Study design eFigure 2. Cerebral performance categories (CPC) and modified Rankin Scales (mRS) at 6 months in the two intervention groups [file jamaneurol-e202264-s002.pdf]

Supplementary Online Content

Rossetti AO, Schindler K, Sutter R, et al. Continuous vs routine electroencephalogram in critically ill adults with altered consciousness and no recent seizure: a randomized clinical trial. Published online July 13, 2020. *JAMA Neurol*. doi:10.1001/jamaneurol.2020.2264

eTable 1. Exploratory analysis of primary outcome (mortality at 6 months) and functional outcomes

eFigure 1. Study design

eFigure 2. Cerebral performance categories (CPC) and modified Rankin Scales (mRS) at 6 months in the two intervention groups

This supplementary material has been provided by the authors to give readers additional information about their work.

© 2020 Rossetti AO et al. *JAMA Neurology.*

Table S1: Exploratory analysis of primary outcome (mortality at 6 months) and functional outcomes across cEEG versus rEEG in patients with and without hypoxic-ischemic encephalopathy. (Poisson regression models for categorical variables [mortality] and linear regression models for continuous variables [Δ mRS and CPC]). Bold values are significant.

Patients with hypoxic-ischemic encephalopathy	**rEEG (n=52)**	**cEEG (n=60)**	**Crude**			**Adjusted for CCI**		
	**Number**	**Number**	**Relative risk**	**95% CI**	**p-value**	**Relative risk**	**95% CI**	**p-value**
**Mortality at 6 months**	29 (55.8%)	39 (65.0%)	1.16	0.86-1.58	0.327	1.28	0.94-1.75	0.122
	**median (range)**	**median (range)**	**Regression coeff.**	**95% CI**	**p-value**	**Regression coeff.**	**95% CI**	**p-value**
**Δ mRS at 6 Months (survivors)**	1 (-1 – 4)	1 (-1 – 4)	0.39	-0.45-1.22	0.355	0.34	-0.52-1.20	0.432
**CPC at 6 Months (survivors)**	1 (1-3)	2 (1-3)	0.15	-0.37-0.68	0.560	0.21	-0.32-0.75	0.428
Patients without hypoxic-ischemic encephalopathy	**rEEG (n=130)**	**cEEG (n=122)**	**Crude**			**Adjusted for CCI**		
	**Number**	**Number**	**Relative risk**	**95% CI**	**p-value**	**Relative risk**	**95% CI**	**p-value**
**Mortality at 6 months**	59 (45.4%)	50 (41.0%)	0.90	0.68-1.20	0.483	0.90	0.68-1.20	0.466
	**median (range)**	**median (range)**	**Regression coeff.**	**95% CI**	**p-value**	**Regression coeff.**	**95% CI**	**p-value**
**Δ mRS at 6 Months (survivors)**	1 (-5 – 4)	1 (-3 – 5)	0.73	0.10-1.36	**0.023**	0.74	0.12-1.35	**0.019**
**CPC at 6 Months (survivors)**	2 (1-4)	2 (1-4)	0.06	-0.24-0.35	0.710	0.06	-0.24-0.35	0.706

Legend: rEEG = routine electroencephalography; cEEG = continuous electroencephalography; CCI = Charlson comorbidity index; CI = confidence interval; CPC = Cerebral Performance Category; Δ mRS = evolution of modified Rankin Scale between pre-hospitalization and at 6 months.

© 2020 Rossetti AO et al. *JAMA Neurology.*

Figure S1: Study design. cEEG=continuous EEG; rEEG=routine EEG.

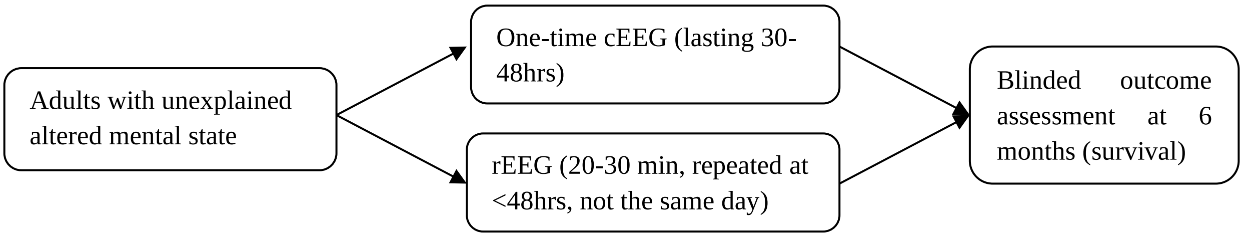

Figure S2: Cerebral performance categories (CPC) and modified Rankin Scales (mRS) at 6 months in the two intervention groups.

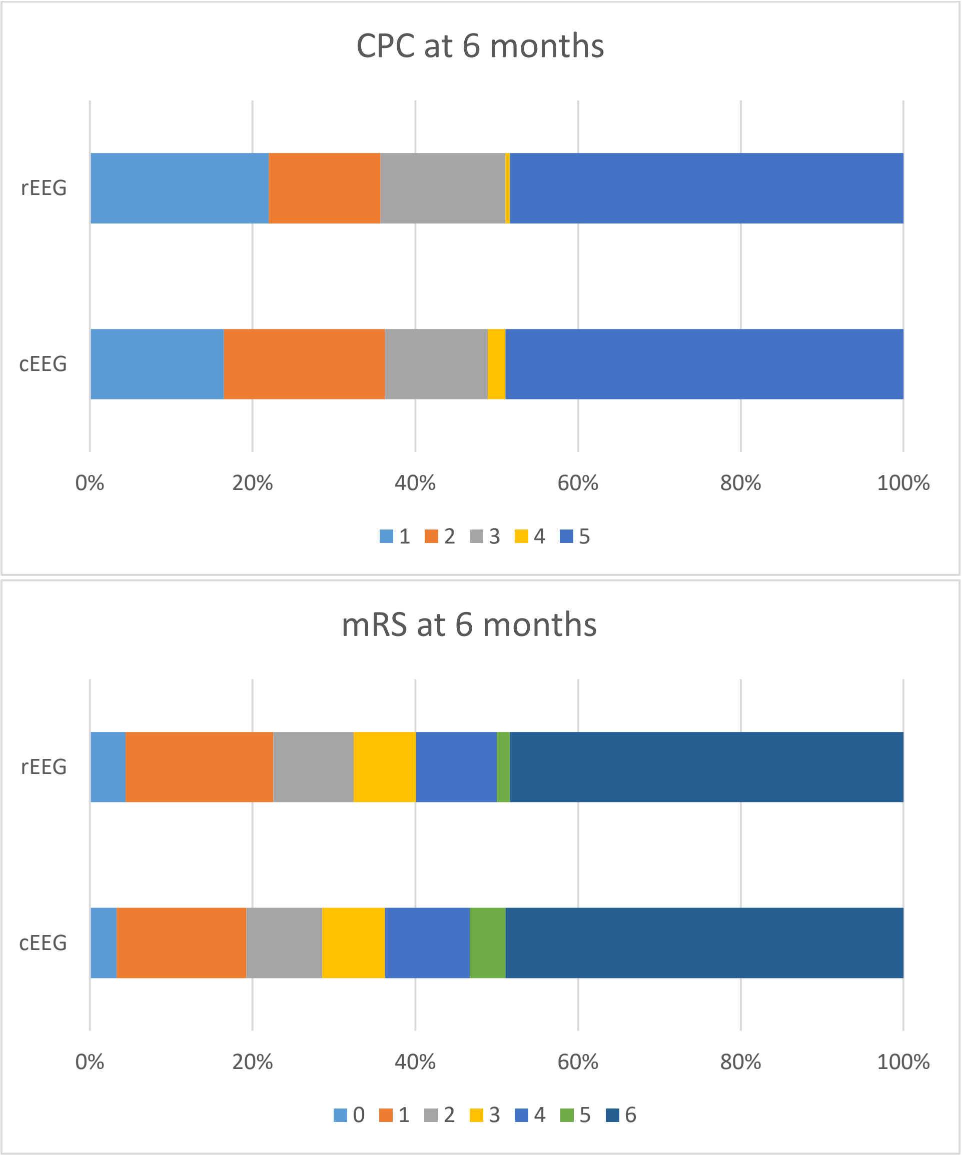

© 2020 Rossetti AO et al. *JAMA Neurology*.
