# Supplementary material for: Continuous vs Routine Electroencephalogram in Critically Ill Adults With Altered Consciousness and No Recent Seizure: A Multicenter Randomized Clinical Trial
Source: JAMA Neurol. 2020 Jul 27;77(10):1–8. doi: 10.1001/jamaneurol.2020.2264 (PMC7385681; doi:10.1001/jamaneurol.2020.2264)
Supplement: Supplement 3. — Data Sharing Statement [file jamaneurol-e202264-s003.pdf]

Data Sharing Statement

Data
Data available: Yes
Data types: Deidentified participant data
How to access data: andrea.rossetti@chuv.ch
When available: With publication

Supporting Documents
Document types: None

Additional Information
Who can access the data: qualified researchers whose proposed use of the data has been approved
Types of analyses: any purpose related to the aim of the study
Mechanisms of data availability: signed data access agreement (also including IRB aproval)
Any additional restrictions: None
